# A Review on Reinforcements and Additives in Starch-Based Composites for Food Packaging

**DOI:** 10.3390/polym15132972

**Published:** 2023-07-07

**Authors:** Pedro Francisco Muñoz-Gimena, Víctor Oliver-Cuenca, Laura Peponi, Daniel López

**Affiliations:** Instituto de Ciencia y Tecnología de Polímeros (ICTP-CSIC), C/Juan de la Cierva 3, 28006 Madrid, Spain; pfmunoz@ictp.csic.es (P.F.M.-G.); victor.oc@ictp.csic.es (V.O.-C.)

**Keywords:** starch, nanocomposite, biopolymers, biobased, nanoparticles, active packaging, reinforcement, additives

## Abstract

The research of starch as a matrix material for manufacturing biodegradable films has been gaining popularity in recent years, indicating its potential and possible limitations. To compete with conventional petroleum-based plastics, an enhancement of their low resistance to water and limited mechanical properties is essential. This review aims to discuss the various types of nanofillers and additives that have been used in plasticized starch films including nanoclays (montmorillonite, halloysite, kaolinite, etc.), poly-saccharide nanofillers (cellulose, starch, chitin, and chitosan nanomaterials), metal oxides (titanium dioxide, zinc oxide, zirconium oxide, etc.), and essential oils (carvacrol, eugenol, cinnamic acid). These reinforcements are frequently used to enhance several physical characteristics including mechanical properties, thermal stability, moisture resistance, oxygen barrier capabilities, and biodegradation rate, providing antimicrobial and antioxidant properties. This paper will provide an overview of the development of starch-based nanocomposite films and coatings applied in food packaging systems through the application of reinforcements and additives.

## 1. Introduction

The use of plastics is essential to many sectors of our economy [1] due to their low production costs, durability, good mechanical properties, and resistance to oil and most chemicals. Consequently, the food packaging sector is the sector with the highest percentage of plastic use [2]. Although the use of non-biodegradable plastics is a matter of concern in society and multiple industries [1], the accumulation of the residues produced by these plastics is a critical problem for the environment, causing several problems in our daily lives and affecting nearly every ecosystem. Moreover, plastics pose a threat to oceans, wildlife, public health, and economic sectors that directly depend on marine wildlife health [3,4,5].

Therefore, to minimize the impact of plastics, it is vital to develop new polymeric matrices that are biodegradable and eco-friendly. Polymers derived from renewable sources, such as starch, cellulose, or chitin, are an excellent approach to fulfill these conditions. However, the current transition rate from petroleum-based polymers to biodegradable polymers is rather low and still inconsistent. Companies prefer to recycle already-used plastics instead of investing in new polymer matrices due to the poorer mechanical and barrier properties that these newly developed matrices show [6,7].

**Figure 1:**
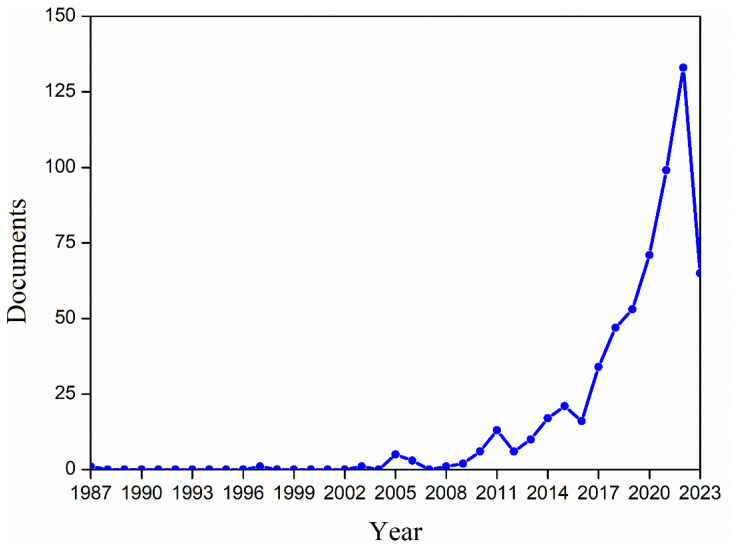
Analytics related to keywords: starch, active, and packaging (source: Scopus).

To improve these limiting properties, a push toward the research and development of new and suitable matrices has to be made. In this context, starch-based polymers rise as one of the most promising alternatives to traditional petroleum-based polymers in the food packaging sector. The growing interest in sustainable plastics can be seen in Figure 1 by the overwhelming increase in research papers and reviews published dealing with the topic: since 2003, almost 600 scientific papers have been published, of which 559 were published after 2013, and 132 during the last year, 2022 (source: Scopus; keywords: starch, active and packaging). The significant increase in research on this topic highlights the demand for sustainable packaging materials.

Starch is a natural biopolymer extracted from conventional botanical sources such as potatoes, rice, or corn, but can also be extracted from non-conventional resources including roots, skins, and pits from vegetables and fruits, most of which are discarded and thrown away. Therefore, starch is abundant and can give value to what is commonly considered “waste”.

Starch usage has been described for thousands of years, from the ancient Egyptians (~4000 BC) and later the Romans using it as an adhesive or the ancient Greeks incorporating it in medical preparations [8]. Nowadays, starch applications range from ingredients in multiple food products (e.g., canned goods, bakery products, baby food, soups and creams, sauces, and dressings) to adhesives, aerogel, films, or edible coatings in the textile, pharmaceutical, paper, packaging, and cosmetic industries [9]. Moreover, starch is considered one of the most promising sustainable materials due to its abundance and excellent biodegradability, with new applications being developed across multiple sectors: green chemistry, water treatments, medical applications (tissue engineering, controlled drug release, and medical dressings) and flexible electronics (supercapacitors, sensors, and conductive substrate films) [10]. However, since native starch granules do not dissolve in cold water, transformation into de-structured thermoplastic starch (TPS) is needed for most starch-based applications [11]. As an example, in the food sector, gelatinization through cooking or processing greatly increases starch digestibility, as the disruption of the ordered structure of gelatinized starch allows easy access of digestive enzymes to starch molecules [12]. 

In the packaging sector, thermoplastic starch (TPS)-based films not only match the properties shown by conventional petroleum-based polymer films, but in some cases, they show a superior capacity to form colorless, tasteless, and translucent layers [13] as well as having excellent gas barrier properties against oxygen and carbon dioxide [14]. TPS can be obtained through two methods: the casting solution method and the extrusion process. Nevertheless, starch films have multiple drawbacks: high hydrophilic behavior, which makes them difficult to use in contact with food that has a high water content, and lower mechanical and water vapor properties compared to petroleum-based polymer films [15,16]. Moreover, starch-based films generally present no antimicrobial or antioxidant properties by themselves. To enhance performance and processability, frequently used strategies include using polymer blends, coating articles with high-barrier materials, using multilayered films containing other films, chemical modification of natural biopolymers, and producing composite films using different fillers [17].

Composite films consist of two phases: a continuous polymeric matrix and a discontinuous filler (organic or inorganic materials with specific shapes) [18]. Moreover, it has been well established that the filler properties and size greatly influence the properties of the nanocomposite, with smaller/nanosized fillers generally having a greater impact [19].

Thus, the objective of this review is to report an interesting scientific discussion on starch-based films for food packaging applications, focusing attention on the reinforcements and additives used and their main effects on starch-based film properties. In particular, after analyzing starch botanical sources and their extraction techniques, the properties of thermoplastic starch are discussed, including common preparation techniques such as solvent casting, extrusion, and film blowing. Then, the most typical organic and inorganic nanofillers used as reinforcements are described, detailing their effects on the mechanical response. Furthermore, additives such as essential oils and functional organic molecules such as carvacrol, eugenol, and cinnamic acid are reported to focus on their antioxidant and/or antimicrobial properties and their application in smart packaging. In the end, a short discussion of their perspective is provided from a circular economy point of view.

## 2. Starch

### 2.1. Starch Sources and Extraction

**Figure 2:**
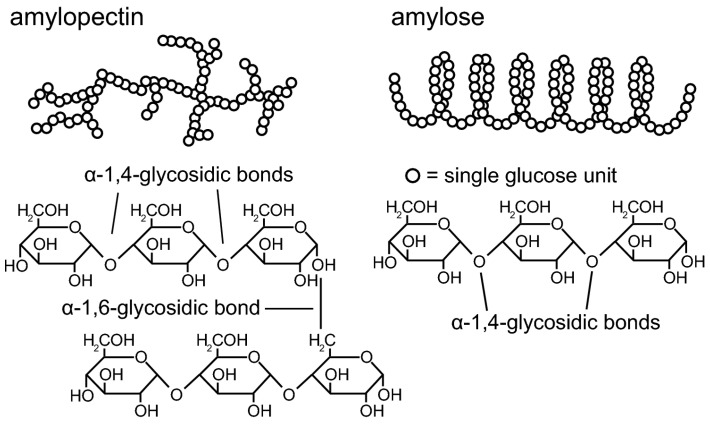
Chemical structure of starch-constituting natural polymers amylopectin and amylose [23].

The structure of starch is formed by the succession of α-D-glucose linked to each other through α (1 → 4) glycosidic bonds [20]. Two different structures are found inside starch matrices: amylose, which has a non-branched, lineal, helical structure with only α (1 → 4) glycosidic bonds, and amylopectin, which has a highly branched structure with 24 to 30 residues of glucose units, as seen in Figure 2. This structure comes as a result of the presence of α (1 → 6) glycosidic bonds, which are responsible for the branched structure, as well as α (1 → 4) glycosidic bonds, like amylose [21]. The ratio between both components fluctuates due to multiple factors, the most important being the source of extraction of starch or even the conditions of the vegetable source. In general, most native starches contain 20–30% amylose and 70–80% amylopectin [22].

Starch can be extracted from multiple sources and, as a result, the ratio between amylose and amylopectin as well as the characteristics and properties of the obtained native starch can vary. Several methods have been used for starch extraction, of which two are the most commonly used. In the first method, starch extraction is carried out after treatment with a dissolution of hydrogen sulfite 0.1 M at 50 °C for 24 h, after which the substrate is ground in a laboratory grinder with distilled water to extract all the starch. After several screenings through different mesh sizes, the starch slurry is centrifuged several times and then dried in an oven for 12 h at 40 °C [24]. In the second method, starch is obtained through treatment with an aqueous solution of sodium metabisulfite 0.1 M for 30 min, after which the subtract is ground with distilled cold water in a laboratory grinder. The homogenate is then screened through different mesh sizes and then the filtrate is kept undisturbed until the complete precipitation of starch which is recovered and treated with an aqueous solution of NaOH 0.2 M for 1–2 h and successive washes with distilled water, and finally dried at 40 °C for 12 h [25]. Among the many possible botanical sources for starch extraction, Table 1 shows the main characteristics of three of the most common starch sources: corn, potato, and rice.

**Figure 3:**
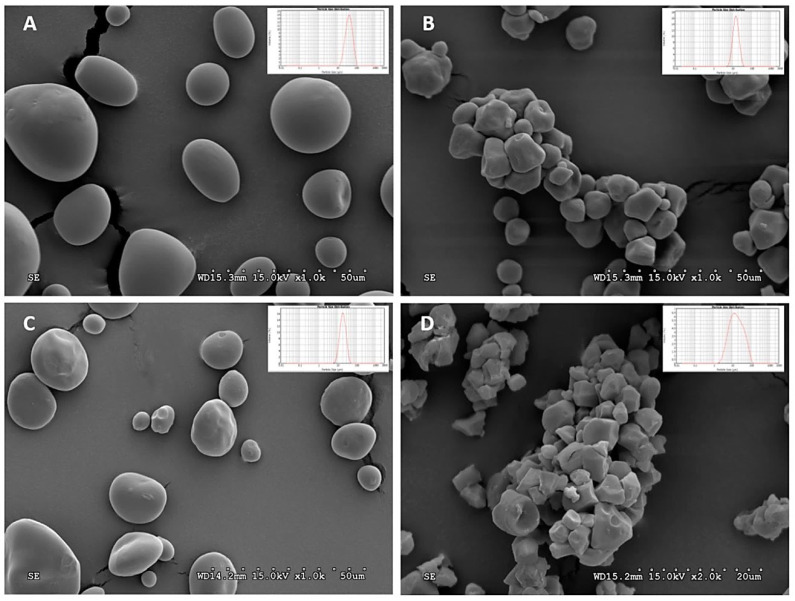
SEM images of various starches: (**A**) potato (1000×), (**B**) corn (1000×), (**C**) wheat (500×), and (**D**) rice (2000×) [39].

The shape and size of the starch granules are characteristic of the source from which the starch was extracted. In corn, starch granules are angular shaped with a diameter of 11.5 ± 4.3 µm; potato starch granules exhibit large irregular and oval or cuboidal forms, with typical granule size for small and large potato starch granules between 1 and 20 µm. Meanwhile, rice starch granules present pentagonal and angular forms with a size of 3 to 5 µm [37]. A wide variety of starch botanical sources and their granular forms is reported in Table 2 and Figure 3.

**Table 2:** Size and shape of starch granules from different botanical sources [38].

Plant Source	Size (µm)	Shape
Maize	2–30	Round and polyhedral
Waxy maize	2–30	Round and polyhedral
Wheat	10 and 10–30	Discs
Waxy wheat	>10	Spherical and lenticular
Rice	<20 and 2–8	Polygonal and angular
Tapioca	5–45	Spherical/lenticular
Cassava	30	Kettle-drum truncated
Potato	<110	Oval and irregular
Sweet Potato	1–100	Oval, spherical, round
Pea	2–40	Oval, spherical, round
Lotus	14–31	Oblong

### 2.2. Thermoplastic Starch, TPS

**Figure 4:**
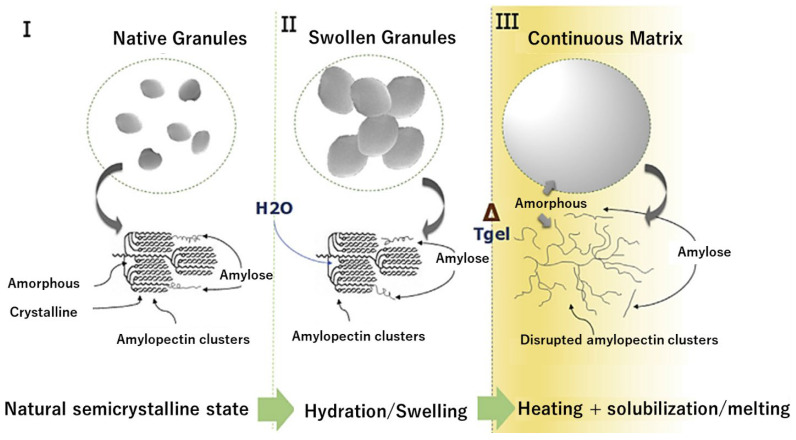
Illustrative scheme of starch granule disruption [42].

Starch has received significant interest in the form of a thermoplastic polymer, namely thermoplastic starch, TPS. Even though starch is a poor choice to replace plastic as it does not exhibit true thermoplastic behavior, it can be transformed into a continuous polymeric-entangled phase through the addition of sufficient water and/or plasticizers [40]. During the processing of TPS, various physical and chemical reactions are involved such as water diffusion, expansion of granules, gelatinization, melting, and crystallization [41]. The disruption process of starch to form thermoplastic starch requires generating an amorphous matrix by the complete or partial disintegration of the granular architecture. In particular, starch conversion into TPS in the presence of water follows a three-step process: granular swelling, amylopectin cluster breakage, and amylose/amylopectin solubilization, as seen in Figure 4 [42]. During the first stage, the absorption of water in its amorphous domains swells the starch granule. This process is reversible when the temperature remains low; however, if the temperature exceeds the gelatinization temperature (Tgel), the granular structure starts to collapse. The specific Tgel is a characteristic property of each starch, linked to the branch chain length of amylopectin, with values for common starches ranging between 50 and 85 °C [43]. However, gelatinization is a complex process, as it depends on multiple variables including botanical source, water content, and composition (amylose/amylopectin ratio, protein, lipids, ash content, etc.), as well as morphology and size of the starch granules [44]. For instance, under differential scanning calorimetry (DSC) study, a single peak is observed when the gelatinization water content is in an excessive amount, while multiple peaks and bumps can be found from gelatinization thermograms when the water content is below, e.g., 60% [45]. On a similar note, amylose content has been reported to both positively and negatively influence the gelatinization temperature [44,46]. 

The glass transition temperature (Tg) of starch is substantially higher than its decomposition temperature [47], thereby impeding its potential usage as a bioplastic. To attain the capacity to process native starch, it is essential to utilize plasticizers, small molecules with low volatility, to decrease the melting point. Additionally, the inherent brittleness, inadequate mechanical properties, and barrier properties of starch-only films pose a significant challenge in their handling. During the thermoplastic process, water contained in starch and the addition of plasticizers (such as glycerol or sorbitol) decrease the internal starch hydrogen bonding [48], increasing the flexibility of the resultant films and improving the barrier and sorption properties of films, causing better packaging qualities [49].

The process of generating a starch film initiates with the application of heat to starch granules in an excess of water, thus facilitating the creation of a viscous solution. The viscoelastic behavior of TPS can be classified according to the quantity of plasticizer present: a glassy profile (hard and stiff but brittle) at low plasticizer concentrations, followed by a rubbery behavior (flexible) characterized by a glass-to-rubber transition as plasticizer content increases, concluding in a gel-like state at very high plasticizer content [50]. The microstructure, particle size, and roughness of the starch source influence the viscosity of the film-forming suspension, promoting a modification of the filmogenic matrix after the drying process, which affects the physicochemical and mechanical properties of the resulting film [51,52].

TPS exhibits a few limitations in its utilization, including: a significant hydrophilic nature, making it susceptible to water; relatively poor mechanical attributes when compared to conventional polymers; and a significant variation in post-processing properties [53]. In addition, retrogradation and crystallization of the mobile starch chains lead to an undesired change in thermomechanical properties [54]. Susceptibility to humidity can be observed in a typical plasticized TPS model system. Under a low humidity environment, stability is adversely affected due to the diffusion of plasticizer out of the TPS matrix, while during high humidity conditions, water diffuses into the product. Consequently, the loss of plasticizer in a low-humidity environment prompts brittleness, whilst the excess absorption of moisture in a high-humidity environment causes shape- and texture-retention concerns [55]. The relative air humidity and the plasticizer type and concentration affect the film water vapor permeability (WVP) [56]. Another undesired effect is starch retrogradation, which is a phenomenon used to describe the stiffening and decreased extensibility of TPS materials over time, linked to the association of dissociated amylose and amylopectin chains in a gelatinized starch dispersion forming more ordered structures [51].

### 2.3. Preparation of Starch-Based Films

There are three main techniques for the preparation of starch-based films; the casting procedure, the extrusion/thermocompression process, and the blow molding approach, which are briefly discussed in this review. Additionally, other alternative processing techniques based on the plastic industry have also been used for the elaboration of starch films, including injection molding, compression molding, foaming, and spinning (melt-, wet- and electro-spinning) [57].

#### 2.3.1. Solvent Casting

The solvent casting technique is by far the most popular in starch film production and has been widely documented in the academic literature. The technique was developed at the end of the 19th century to manufacture thin plastic films for the emerging industry of movies and photography [58].

The casting of starch-based films typically includes four steps: solution preparation, gelatinization, casting, and drying. As a general method [59,60], the starch and plasticizers are mixed in water and heated to 80–95 °C for 10–15 min while under constant shaking. The gelatinized suspensions are poured on Teflon or acrylic plate and dried in an oven (40–75 °C) for 24 h to constant weight. The film thickness, usually 0.02–0.10 mm, can be adjusted by the amount and concentration of the starch suspension poured into the mold.

Despite its simplicity and popularity, the long drying phase makes this method slow and discontinuous, producing small quantities of material after a long process. Moreover, the casting methodology presents certain drawbacks: difficulty when combining materials of different natures; separating the film from the underlying support can be challenging; and limited capacity of scaling up the technique from an experimental setup to industrial production [61].

#### 2.3.2. Tape Casting

Tape casting was first introduced by Glenn Howatt in the 1940s, to produce capacitor dielectrics, and, to this day, continues to be one of the main applications for this technology [62]. The technique can be seen as an upgraded version of the solvent casting method, as it has the potential to facilitate researchers utilizing the classical casting method to scale up production and create biopolymeric films with significantly larger dimensions. However, there is very limited literature about the utilization of tape casting on starch-based films, with the majority elaborated by the same research group, and the method is probably limited by the difficult technological transfer from other industries [63].

**Figure 5:**
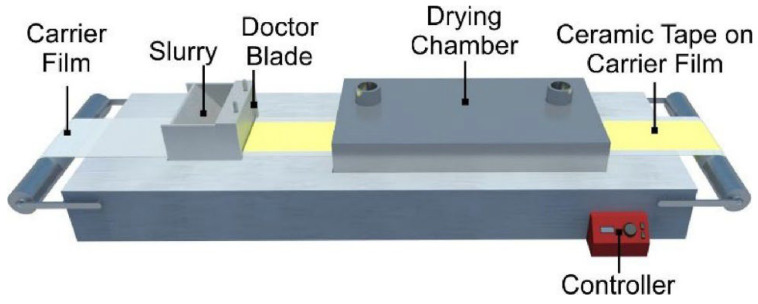
Representation of a tape casting setup used for ceramic manufacturing [67].

The tape casting method allows the casting of the film-forming solution on large supports (batch process) and on continuous belts (continuous process), where the film thickness can be modulated by an adaptable blade positioned at the base of the spreading device [64], as seen in Figure 5. The support where the film suspension is placed permits a regulated drying via heat conduction, hot air circulation (heat convection), and/or infrared radiation; with a consequent reduction in the final thickness, usually ranging between 20 μm and 1 mm [65]. Scheibe et al. [66] successfully tape-casted starch-glycerol-fiber films and starch-glycerol-cellulose fiber bags with different properties by changing the film thickness.

#### 2.3.3. Extrusion

From an industrial standpoint, the manufacturing of TPS demands a highly effective mass-production technique; a simple solution is using processes with the standard equipment used in the manufacture of synthetic polymers. Considering this, hot extrusion presents itself as an answer to scale up biopolymeric film production.

Throughout the extrusion process, starch is subject to elevated pressure and temperature, as well as the application of mechanical shear forces, leading to a range of chemical and physical reactions, disintegrating the starch granules [68]. The plasticization and melting of TPS can be facilitated through the heating and shearing effects resulting from the rotation of internal screws within the barrels utilizing electric heating, where temperature manipulation can be employed across various zones within cylindrical barrels to attain desired outcomes relating to mixing, flow, pressure, and flow rates [69].

González-Seligra et al. [70] studied the effect of screw speed on starch-glycerol-water mixes extruding under the same temperature profile and process conditions. A slow screw speed (40 rpm) resulted in high non-gelatinized broken grains and uneven glycerol distribution, leading to high crystallinity and low deformation resistance. Extrusion at 80 rpm was ideal for homogeneous TPS in threads and films but presented accelerated retrogradation. Finally, high speed extrusion (120 rpm) achieved stronger amorphous TPS films with slower retrogradation. On a similar note, Li et al. [71] studied the effect of modifying amylose content on extruded TPS films. The study found that processability decreased with increased amylose content, but the effects could be countered by modifying the extrusion parameters. Higher amylose films presented better mechanical properties and high impact strength. Moreover, the glass transition temperature (Tg) of the extruded films was studied using DMA, and showed a rising trend with increasing amylose content, attributed to the different molecular sizes of amylose and amylopectin and their phase transitions.

#### 2.3.4. Film Blowing

The process of film blowing is a technique frequently used to manufacture self-supporting plastic films, which involves extruding a hollow tube and then expanding it into a bubble by raising the pressure within the tube [57]. The popularity of film blowing in plastic film production is due to its simple production equipment, low investment, continuous production, and adjustable film size [72].

Rejak, A. and Mosciki, L. [73] processed different starches through film blowing and found maize and wheat starch to be unfit for packaging due to their brittle, opaque and thick films obtained. However, potato starch films led to semi-transparent films amenable to blow-molding. Furthermore, the addition of emulsifiers further improved film flexibility and strength. On a similar note, Dang & Yoksan [74] examined the effect of replacing 50% of glycerol with larger-molecular plasticizers, xylitol or sorbitol, in TPS blown films. While the glycerol-only films presented higher melt flowability and elongation at break, and lower Tg, they also displayed stickiness and shrinkage. Meanwhile, the mixed plasticizers achieved significant tensile strength, stiffness, water vapor barrier and oxygen barrier properties, and dimension stability improvements.

Nonetheless, TPS production by film blowing may result to be complex due to the low melting tenacity of starch [57]. Therefore, a frequent approach to ease the processability and mechanical properties of the resulting films is the preparation of starch blends combined with other biodegradable polymers, such as PLA [75], PCL [76], PBAT [77,78] or chitosan [79].

## 3. Nanoparticles

The hydrophilic characteristic of starch results in elevated water vapor permeability (WVP) and low tensile strength and Young’s modulus in contrast to synthetic materials of manufactured products. A feasible approach to enhance these attributes involves the integration of reinforcement fillers to create starch-based nanocomposites.

The incorporation of nanoparticles as fillers in polymers is a widely recognized technique to enhance the properties of polymeric materials while using less product. For an equal amount of filler, if the dispersion exhibits homogeneity, the influence on the macroscopic characteristics will be more pronounced for nanoparticles of a smaller size, attributable to the augmented polymer/filler interface [80]. However, the aggregation of nanoparticles may result in an adverse impact on the modulus and strength of the material as a consequence of the formation of rigid regions within the polymer matrix and a decline in the interfacial surface area [81]. Several techniques are often used to obtain a complete and homogenous dispersion of NPs in the polymeric matrix: mechanical mixing (i.e., high-speed shear milling, three-roll milling, and high-pressure homogenizing) or ultrasound sonication [82,83].

**Figure 6:**
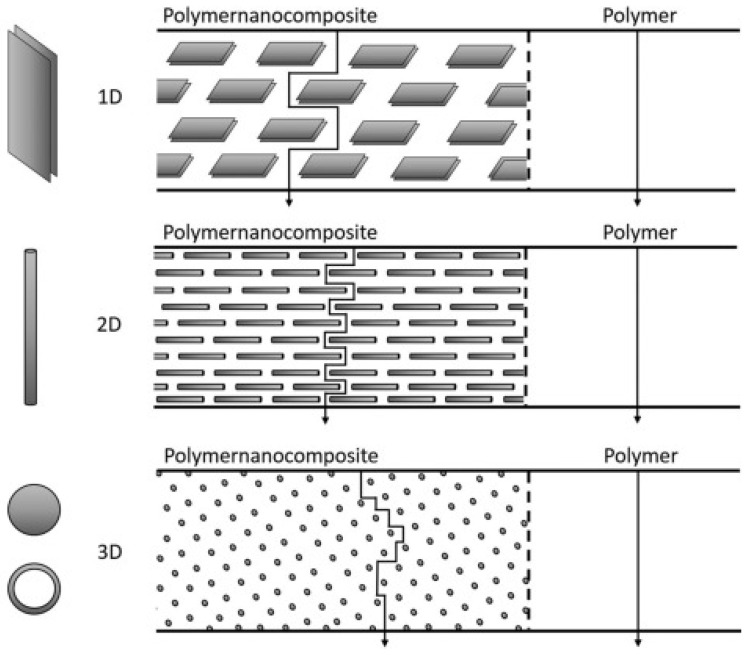
Tortuosity pathway in polymeric matrices with different geometric nanoparticles [87].

Nanoparticles can be grouped by their nature into inorganic nanofillers (e.g., metal and metal oxide nanoparticles, carbon nanotubes, and nanolayered clays) and organic nanofillers (e.g., cellulosic whiskers, lignocellulosic materials, chitin whisker, and starch nanocrystal) [84]. Alternatively, nanoparticles can also be classified into three main categories according to how many of their dimensions are on the nanometric scale and according to their geometry [84,85,86], as seen in Figure 6.

Recently, Wolf et al. [84] compared hundreds of nanocomposite studies in an attempt to compare the tortuosity effect of different nanoparticle shapes on barrier properties. While the review did confirm that layered nanoparticles were more efficient than isodimensional and elongated nanoparticles, no clear trend was established on the selective transport mechanisms.

As nanotechnology innovations make their way into different industrial sectors, many countries are establishing controls over the use of nano-based products. However, regulation frameworks applied to control and ensure the safe use of nanotechnologies differ across countries and regions [87]. For example, TiO_2_ has approval for use in human food, medicines, cosmetics, and food contact products by the US FDA [88]. However, due to the potential genotoxicity of TiO_2_ NPs, the European Commission in 2022 banned the use of TiO_2_ as a food additive and encourage its replacement in medicines over health safety concerns [89].

In the EU, nanomaterials are covered by the same regulatory framework that ensures the safe use of all chemicals and mixtures, i.e., the REACH and CLP regulations. The EU has added regulatory measures for nanomaterials in the food and feed chain, together with risk assessment guidance. Current EU legislation includes Regulation (EC) No. 1169/2011 on the provision of food information to consumers, which defines ‘engineered nanomaterial’ as any intentionally produced material with at least one or more dimensions of the order of 100 nm or less, including structures, agglomerates and aggregates which may exceed this size but retain properties of the nanoscale [90]. EU Regulation on Plastic Food Contact Materials and Articles (10/2011) sets an overall migration limit (OML) and specific migration limits (SML). OML is set at 60 mg (of substance) per kg (of food or food stimulant) to guarantee that packaging materials do not transmit excessive amounts of compounds which, even if they are not hazardous, might cause an undesirable change in the composition of the food [91]. As an example of a safety assessment, the study on silver nanoparticles (Ag NPs) for use in food contact materials [92] determined that Ag NPs remained embedded in the polymeric matrix and did not pose a toxicological threat. In ionic form, silver did present low levels of migration which did not exceed the proposed acceptable daily intake (ADI) of 0.9 µg/kg per day. Nevertheless, the study highlights that estimated exposure combined with other dietary sources already exceeded the set ADI limit.

### 3.1. Inorganic Nanofillers

Incorporating inorganic nanoparticles is a highly effective form of enhancing the mechanical and physical properties of starch-based films. The incorporation of these additives in polymeric nanocomposite causes an improvement in mechanical and barrier qualities, including enhanced gas barrier qualities, and stability across a range of temperature and moisture levels [93]. Furthermore, some of them can also add new functional properties to these matrices including antimicrobial properties by reducing the growth of microorganisms [94]. There are many different types of fillers in this category, including metal nanoparticles, metal oxides, and nanoclays.

#### 3.1.1. Nanoclays

An interesting class of nanofillers that solves the issues of starch matrices previously mentioned is nanoscale lamellar silicates, commonly referred to as nanoclays. The economic cost, biocompatible nature, and widespread availability of nanoclays have seen them being frequently utilized for the production of biodegradable films and coatings, as well as synthetic films to improve their barrier and thermal stability properties [95]. According to reports [96], the incorporation of nanoclays, even at low concentrations, can produce a reduction in gas permeability through polymer films of 50–500 times. The integration of nanoclays into polymers emerged as a pioneering polymer nanocomposite technology, yielding a novel material for the purpose of food packaging [97], and representing nearly 70% of the market volume of commercial nanoscale applications in the early 2010s [98].

**Figure 7:**
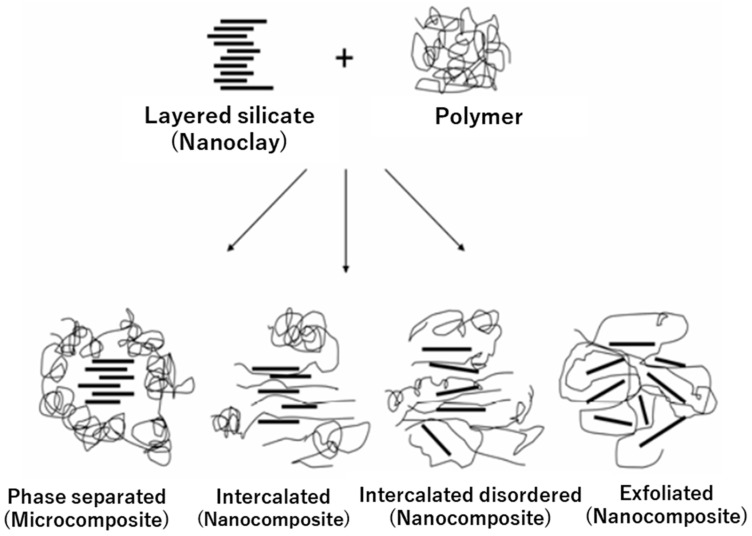
Possible dispersion states of nanoclay in composite structures [100].

Typically, within a polymer matrix, nanoclays can display three distinct morphologies of nanoclay, depending on the dispersion state of the nanoclay, and the degree of separation between the silicate layers, as well as the level of polymer penetration between them. These morphologies are: aggregated, intercalated, and exfoliated [99], as seen in Figure 7. In nanocomposites, the clay layers must be uniformly dispersed in the polymer matrix (intercalated or exfoliated), as opposed to being aggregated as tactoids [100].

The impermeable properties of nanoclays dispersed within the film oblige water molecules to travel through a convoluted path to cross the matrix, improving barrier properties to water vapor [100]. Increasing the aspect ratio and the volume fraction of the nanoplatelets improves the barrier properties of nanocomposites [96]. Several studies have documented nanoclay-based packaging materials employed in conjunction with different active agents to achieve film formation that manifests antimicrobial activity [101]. Moreover, the enhanced tortuosity impact of dispersed nanoclays effectively prolongs the retention or regulates the transfer of antibacterial agents through polymer matrices.

Osman et al. [102] demonstrated the importance of nanofiller dispersion by contrasting dolomite/starch films versus ultrasonicated dolomite/starch; sonication reduced particle size, increased crystallinity and matrix homogeneity, and significantly improved mechanical properties. On a similar note, Gao et al. [103] tested the effect of five clays with different hydrophilicity levels on the properties of starch-clay nanocomposites by film blowing. All nanoclay-reinforced films presented improved mechanical and barrier properties compared to the control film. While the higher hydrophilic clay presented the lowest WVP, the medium hydrophilic clay exhibited better compatibility and dispersion within the starch matrix, which resulted in the highest tensile strength and lowest elongation.

Interestingly, Müller et al. [61] compared two preparation methods: dispersing nanoclays in glycerol for 24 h and subsequently incorporating this into the starch, versus adding all the ingredients in a single step. While both thermopressed bentonite-starch films presented improved mechanical and barrier properties compared to the control films, the previously dispersed films showed significantly higher reinforcing effects and lower WVP (60% lower than control samples). The procedure led to an enhancement in the interactions between the nanoclays and glycerol, limiting the availability of glycerol and water interactions with starch, reducing hygroscopicity and plasticization. The study demonstrates that the properties of nanocomposite materials are a direct consequence of the interactions that take place between the elements comprising the composites.

##### Montmorillonite (MMT)

Montmorillonite (MMT) is a relatively cheap and widely available natural clay derived from volcanic ash/rocks [98], popular as a reinforcement in polymer composites due to its high surface area, significant aspect ratio and high swelling properties in polar spaces [99,104]. MMT is comprised of two silica tetrahedral sheets that are interconnected to a central octahedral sheet (2:1 layered structure) composed of aluminum or magnesium, where each layer is about 1 nm thick and with a lateral dimension of 100–1000 nm [105]. The hydrophilic nature of natural MMT, which originates from the presence of sodium cations within its interlayer spaces, makes it compatible with hydrophilic polymers like starch [106], as the polar hydroxyl groups of water in the TPS chain strongly interact with the silicate layer of pristine MMT, allowing the polymer chains to intercalate [107].

Integration of MMT NPs as a filler in starch-based films is frequently used to overcome low mechanical resistance and poor barrier properties [108]. Huang et al. [109] presented corn starch/MMT nanocomposites with remarkable mechanical augmentation through the incorporation of 5% nanoclays, specifically in the tensile strength and strain with an increase of 450% and 20%, respectively. Tang et al. [100] compared the influence of incorporating MMT and modified-MMT nanoclays. The starch/MMT composite films exhibited superior mechanical properties and lower WVP compared to both the starch/modified-MMT and the blank reference. Conversely, the modified MMT presented a hydrophobic surface and was, therefore, less miscible in the hydrophilic starch matrix resulting in little or no intercalation or exfoliation.

##### Other Nanoclays

Kaolinite is an abundant 1:1 clay with little research into starch composites; moreover, kaolinite-starch interactions are considered very weak, as kaolinite clay sheets present strong hydrogen bonds complicating dispersion in aqueous solutions and polymeric matrices [110]. However, the use of aprotic polar molecules like DMSO is a usual approach to weaken the clay sheets’ bonds and intercalate kaolinite. Mbey et al. [110,111] studied the DMSO intercalation effect on cassava starch/kaolinite films. The DMSO enhanced nanoclay dispersion and, as a result, films presented augmented transparency, water uptake, and UV barrier effect, while lowering Tg. An interesting next step could be the addition of Ag NPs in the interlamellar space of kaolinite with DMSO by chemical-induced reduction [112], which Girdthep et al. [113] used in PLA films to further increase nanoclay dispersion, enhance gas barrier properties, and provide long-lasting antimicrobial properties.

Ren et al. [114] prepared potato TPS/halloysite nanobiocomposites to study the influence of two plasticizers and the effect of nanoclay content. Halloysite nanoclay increased the thermal stability and reduced the moisture content of films independent of the plasticizer used. Glycerol improved halloysite nanotube dispersion in the matrix more than sorbitol, due to stronger hydrogen bonds, allowing a more obvious enhancement in tensile properties.

#### 3.1.2. Metal Nanoparticles

##### Silver Nanoparticles (Ag NPs)

**Figure 8:**
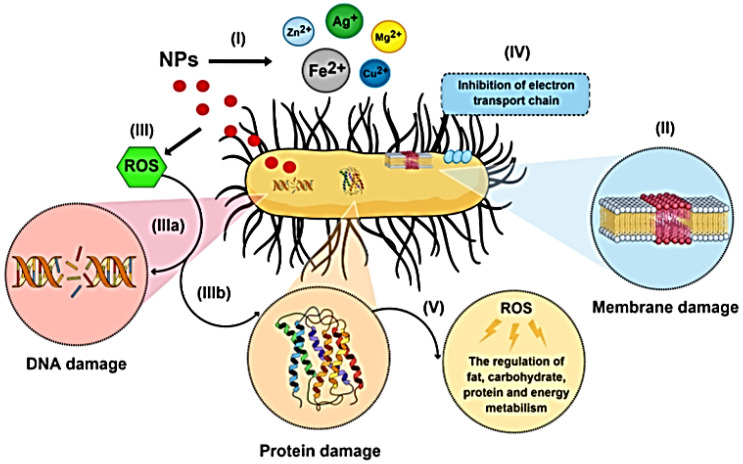
Antimicrobial mechanisms of Ag nanoparticles [120].

Looking at the use of metallic nanoreinforcements, a variety of metal nanoparticles have been incorporated in multiple polymeric nanocomposites for a wide range of applications, for example, gold, copper, silver, palladium, and cobalt [115]; however, silver is by far the most popular. In starch matrices silver nanoparticles are an important additive due to their ability to be synthesized via green chemistry [116,117]. This synthesis also presents the added value of being inexpensive, having a straightforward one-step process, being easily scaled up for large-scale synthesis, and not requiring hazardous chemicals, high pressure, energy, or temperature [118]. Moreover, silver is a well-known antibacterial component that can kill over 650 distinct types of microorganisms, including gram-positive and gram-negative bacteria, fungi, and viruses [118]. The concentration of free Ag^2+^ ions affects the numerous processes that Ag uses. These are the suggested mechanisms:

(1) Ag^2+^ interacts with sulfhydryl groups in the cell wall to generate insoluble compounds that are parts of several enzymes involved in the production of transmembrane energy and the transport of electrolytes.
(2) Ag^2+^ inhibits the cytochrome oxidase and NADH-succinate-dehydrogenase regions of the bacterial respiratory chain.
(3) Ag^2+^ enters the cell and interacts with the bacterial DNA. This reversible binding to the nucleotide bases results in denaturation by breaking the hydrogen bonds between the neighboring purines and pyrimidines [119], as seen in Figure 8.

Ag NPs biosynthesized from plant extracts have demonstrated the capacity to scavenge free radicals in addition to their antibacterial capabilities. This is primarily because phenolic chemicals are present in the structure of Ag NPs [121].

In thermoplastic starch matrices for food packaging Ag nanoparticles have been used as an important additive highly increasing the antimicrobial properties of the matrix, more than essential oils and nano-emulsions. Multiple edible films containing starch as a matrix and essential oil or silver nanoparticles were obtained via solvent casting. The antimicrobial activity of these films was measured proving that edible films containing silver nanoparticles showed almost a three-times-bigger diameter of the inhibition zone [122]. The antioxidant capacity in starch matrices has not been proved yet; nevertheless, Ag Np greatly increases mechanical properties, as explained in a recent study [123] where Ag NP/starch edible films showed a great increase in the physical resistance of films. Khurshid et al. [124] proposed a green approach for the synthesis of Ag NPs from *S. asoca* leaf extract; the obtained Ag NP/starch films exhibited lower water solubility and WVP with impending antibacterial activity. The addition of Ag NPs to starch and chitosan coatings on paper surfaces upgraded the barrier and mechanical properties and repressed the growth of *S. enteritidis* and *E. coli* [125]. Similarly, Ag NPs were dispersed in mushroom starch/agar films to increase antibacterial activities, water vapor properties and hydrophobicity [126].

##### Zinc Oxide (ZnO) NP

Compared to other metal oxide NPs, ZnO has received the highest attention from researchers. Among its many attributes are biocompatibility, nontoxic, chemically stable under exposure to both high temperatures and UV light, and the recognition by the US Food and Drug Administration (FDA) as a generally recognized as safe substance (GRAS) [127,128].

ZnO NPs’ inclusion in buckwheat starch films enhanced physical and water barrier qualities as well as their antimicrobial activity. The 3% ZnO NPs film in particular showed antibacterial action against *L. monocytogenes* and preserved the quality of fresh-cut mushrooms throughout storage [129]. Anugrahwidya et al. [130] successfully employed ZnO NPs to increase the tensile strength of starch films and the shelf life of bread. Degradation tests in soil and seawater showed NPs slightly delayed degradation. However, the addition of chitosan to the film matrix resulted in even higher mechanical properties and faster degradation.

In another study, ZnO nanoparticles and fennel essential oil (FEO) were combined on potato starch films produced by the casting method. The study reveals an improvement in barrier properties, a decrease in water vapor and oxygen permeability, and tensile strength. The antimicrobial activity of starch films was tested against *S. aureus*, *E. coli*, and *A. flavus* with results showing a significant improvement as the concentration of ZnO and FEO increased [131].

Ni et al. [132] developed a cornstarch-based coating with 5% ZnO NPs for food packaging paper with high hydrophobicity and antimicrobial properties against *E. coli*., and with ZnO migration below the overall migration legislative limits; however, ZnO NPs over 7% led to sharply decreased transparency. In a similar manner, mangos were directly coated with starch and ZnO NPs to prevent post-harvest degradation caused by *C. gleosporides*, prolong shelf life, and preserve the quality of the fruit while being stored [133].

##### Titanium Dioxide (TiO_2_) NP

TiO_2_ is commonly used as a white pigment in the paint and coating industries due to its high refractive index and low absorption, resulting in a high light scattering that renders the materials opaque with outstanding thermal stability [134]. In the packaging sector, TiO_2_ NPs have been used to modify the properties of different biopolymer films including starch-based nanocomposites [135]. In addition, TiO_2_ has antimicrobial properties that destroy microbial cells through the production of active oxygen species (ROS) under adequate light energy [136], which can eliminate pathogenic microorganisms, including bacteria, fungi, and viruses. The antimicrobial activity of TiO_2_ has been widely tested on a variety of microorganisms, including *E. coli*, *S. aureus*, *P. putida,* and *L. innocua* on multiple surfaces [132,137].

Tunma et al. [138] incorporated 0.01% *w*/*w* TiO_2_ into starch films and managed to extend the shelf life of fresh fruits over petrol-based films. Moreover, the composites decomposed under 14 days leaving no trace of NP residue in the soil. However, higher concentrations of NPs led to brittleness and fracture of the packaging. Ostafinska et al. [139] compared the addition of TiO_2_ NPs and titanium nanotubes in TPS films using a combination of solvent casting and melt mixing procedures to increase dispersion. Although individual nanotubes were smaller than the TiO_2_ NP, they tended to form big agglomerates, whereas TiO_2_ NPs exhibited better dispersion and higher interface surface than agglomerated titanium nanotubes, and as a result, the highest increase in shear moduli.

Arezoo et al. [140] combined cinnamon essential oil (CEO) and TiO_2_ NPs in sago starch-based film which presented excellent antimicrobial activity against *E coli*, *S. typhimurium,* and *S. aureus.* On the other hand, the additives caused colliding effects in mechanical and barrier properties, a higher TiO_2_ NP concentration increased tensile strength and Young’s modulus, and lowered elongation at break, WVP, and oxygen permeability, while CEO had the opposite change. The developed films allowed an increase in shelf life and better preservation of fresh pistachios due to the antioxidant and antimicrobial properties of CEO and TiO_2_ combined.

##### Copper Oxide (CuO)

Copper oxide nanoparticles do not only act as reinforcements for the polymeric matrix. Bumbudsanpharoke et al. [141] studied blends of PBAT and TPS reinforced with CuO NP, obtained via extrusion using 30% glycerol *w*/*w* starch as a plasticizer and different amounts of CuO concentrations (0.05–2 wt%). These nanoreinforced blends present higher mechanical properties (TS increased 29.1% and EB increased 67% over the PBAT/TPS film), increased vapor barrier properties, and an antimicrobial rate of over 99% against *E. coli*, as well as presenting great antimicrobial activity against bacteria (gram-positive and gram-negative bacteria) [142], and also some antimicrobial activity against fungi [143], viruses [144] and algae [145].

Due to their small size and large specific surface area of CuO, nanoparticles have remarkable antibacterial potential because they can interact intimately with cell membranes [146]. Copper ions function by rapidly receiving and giving electrons, having a high redox potential, and showing the ability to damage the structural elements of microbial cells to the point of death [147].

Numerous studies have reported that edible films containing CuO nanoparticles presented superior properties to other films in terms of their antioxidant qualities. For example, Rehana et al. [148] reported a case used in medicine against cancerous activity. In another case, CuO nanoparticles were used as an additive in a chitosan edible film. Reinforced films presented higher thermal stability than neat chitosan films and had excellent antibacterial activity against gram-positive and gram-negative bacteria, as well as antioxidant properties [149]. In another example, nanoparticles were synthesized by a green method, and their antimicrobial and antioxidant properties were measured showing excellent results in antimicrobial and antioxidant properties [150]. In DPPH tests, CuO nanoparticles can transmit their electron density to the free radical situated at the nitrogen atom [151].

When used in thermoplastic starch matrices, they not only produce excellent antimicrobial [141] response but also antioxidant and mechanical properties are increased when working as additives as well as reinforcements, respectively. In this case, an active edible film containing Ag, ZnO, and CuO nanoparticles, or a combination of these nanoparticles, in a starch matrix has been obtained via the solvent casting method. The addition of this additive has proven to increase mechanical properties as well as antioxidant properties [93].

##### Other Metallic NPs

The incorporation of magnesium nanoparticles (MgO NPs) in starch films resulted in increased thickness, enhanced antioxidant activities and antimicrobial activity against *E. coli* and *S. aureus,* and decreased solubility, moisture content, and WVP [152]. In the same way, MgO NPS and tea polyphenols reinforced potato starch films [153] and exhibited strong antioxidant activity and strong antibacterial activity against *E. coli* and *S. aureus* with an inhibition zone diameter of 25.60 mm and 27.50 mm, respectively. Moreover, the lower WVP and oxygen permeability of the reinforced films proved to prolong the shelf life of various fruits, as they reduced weight loss of various fruits, while the reduced enzyme activity and inhibition of the microbial growth on surfaces delayed the decline in firmness.

Zirconium oxide nanoparticles (ZrO_2_ NPs) combined with encapsulated essential oil increased the antioxidant, antibacterial, and mechanical properties of potato starch and apple peel composite films [154]. The study also highlighted that the augmented films expanded the shelf life of quail meat by reducing WVP and moisture content, whereas incorporating a combination of Ag, CuO, and ZnO NPs in lower concentrations enhanced mechanical and antibacterial properties while decreasing toxicity against eukaryotic cells [93].

### 3.2. Organic Nanofillers

The exploitation of organic fillers as additives in packaging materials has primarily been motivated by the imperative to mitigate the toxicity concerns associated with synthetic nanoparticles [149], alongside their inherent renewability, sustainability, and abundance. The production of nanoparticles (starch nanoparticles, cellulose nanofibers, chitosan nanoparticles, etc.) in the food industry is gaining attention due to their controlled release, stability, bioavailability, and ability to deliver active ingredients [155]. The combination of biodegradable polymers as a matrix and bio-based filler provides a wide range of opportunities to develop improved, safe, and environmentally friendly packaging materials.

#### 3.2.1. Starch Nanomaterials

These organic nanomaterials possess a multitude of advantageous properties: renewability, biodegradability, unique morphology, high crystalline orientation, surface chemistry, and rheological tendencies, which have attracted significant attention over the past decades [156]. The addition of starch nanofillers has resulted in significant enhancements in the properties of nanocomposites, including mechanical strength, thermal stability, resistance to moisture, oxygen barrier ability, and biodegradation rate [155].

Currently, several terms have been used to describe starch nanomaterials that coexist and have created confusion among researchers. However, there are two distinct classifications of starch nanomaterials persistently reported in the literature: starch nanocrystals (SNC) and starch nanoparticles (SNP) [157]. SNC are crystalline platelets that are derived from starch granules by eliminating the amorphous elements, typically through acid hydrolysis [158,159]. On the other side, SNPs are characterized by their nanoscale size and varying dimensions, which can come from different sources of starch, exhibit variability in crystallinity, and in some cases, be completely amorphous [159,160].

Starch nanocrystals (SNC) constitute crystalline fragments produced through the disintegration of amorphous sectors in starch granules and exhibit nanometer scale dimensions (<100 nm) in at least one dimension. SNC can be obtained from different sources and multiple approaches, with acid hydrolysis with sulfuric acid being the most frequent procedure. In this method [161,162], starch powder is mixed with a 3.16 M H_2_SO_4_ solution (15% *w*/*v*), at 40 °C under mechanical stirring for 5 days. The remaining suspension is repeatedly washed with distilled water and separated using centrifugation at 10,000 rpm and 4 °C until a neutral pH is reached. Finally, the suspension is submitted to mechanical treatment using an Ultra Turrax to avoid aggregates before being freeze-dried.

**Figure 9:**
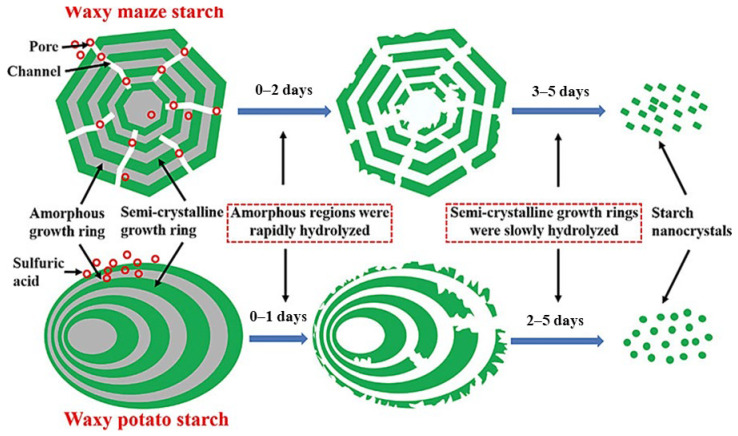
Acid hydrolysis with sulfuric acid of waxy maize and starch [158].

During the initial stage of acid hydrolysis (days 0–2), the amorphous regions, which are more susceptible to acid, are quickly degraded resulting in an increase in crystallinity. The second phase (days 2–5) consists of slow hydrolysis of the semi-crystalline rings, breaking the starch granules into nanocrystals [158,163]. Acid hydrolysis procedures were originally carried out using 2.2 M HCl for longer periods of time [164,165], which resulted in non-charge SNC surfaces compared to the negatively charged SNC prepared utilizing H_2_SO_4_ [166]. When studying the properties of different SNC, Le Corre et al. [167] discovered that botanical origin had a small influence on nanocrystal size, while amylopectin content and molecular structure had a significant effect on the shape and crystallinity; square-shaped SNC were obtained from A-type starches, while round-shaped SNC were generated from B-type starches, as seen in Figure 9.

Nevertheless, the application of SNC generated through traditional acid hydrolysis is restricted by their susceptibility to aggregation, combined with long elaboration periods and low extraction yields [168]. Moreover, the acid hydrolysis approach poses challenges to its implementation in industrial settings due to environmental concerns [169]. In recent years, alternative methods to obtain starch nanomaterials have been proposed, including physical treatments such as ultrasonication [157,170], stirred media milling [171], gamma radiation [172] and high-pressure homogenization [173,174]; enzymatic treatments [169]; nanoprecipitation in ethanol [175,176]; self-assembly methods; and combinations of methods such as acid hydrolysis with ultrasound [168,177], acid hydrolysis with precipitation [178], ball milling with acid hydrolysis [179], or enzymatic treatments with acid hydrolysis [180].

Starch nanomaterials have successfully been used as reinforcement fillers in many natural and synthetic matrices. The incorporation of SNC into starch-based films increased mechanical and water vapor barrier properties [170] and thermal stability [181]. Similarly, mango seed starch-based films, containing mango SNC at a concentration of 5–7.5%, exhibited increased tensile strength and modulus, as well as decreased water vapor permeability. However, the film’s transparency and elasticity were hindered [182]. Some researchers have proposed modification of SNC to increase the effects of the nanofillers. Surface acetylation of SNC improved their affinity to the matrix, increasing the oxygen barrier and mechanical properties of the films [183]. The addition of modified SNP with citric acid by dry precipitation augmented tensile yield strength (from 3.94 to 8.12 MPa) and Young’s modulus (from 49.8 to 125.1 MPa) while lowering elongation (from 42.2 to 35.0%) and WVP (from 4.76 × 10^−10^ to 2.72 × 10^−10^ g m^−1^ s^−1^ Pa^−1^) of starch nanocomposites [184].

#### 3.2.2. Cellulose Nanomaterials

**Figure 10:**
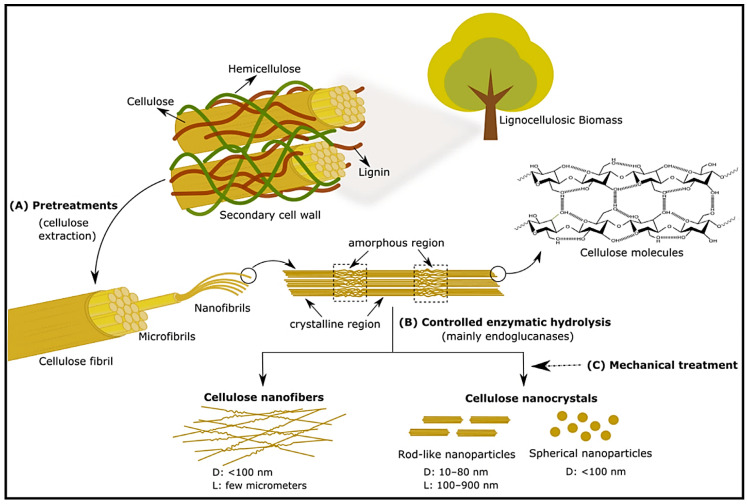
Nanocellulose production through enzymatic hydrolysis. (**A**) Pretreatments for cellulose extraction; (**B**) Controlled enzymatic hydrolysis for production of cellulose nanofibers and nanocrystals; (**C**) Application of mechanical treatment to obtain more uniform particles [196].

Cellulose is the most abundant natural polysaccharide, composed of linear chains of β-1,4 linked D-glucose units, which form strong intramolecular hydrogen bonds which shape its crystalline characteristics [185]. Cellulose serves as the principal structural component of plants, algae, and bacterial organisms, and is considered a valuable resource that can be obtained from various natural sources such as trees, plant stalks, and agricultural residue [186]. In nature, cellulose exists as an aggregation of distinct cellulose chain-forming fibers known as protofibrils, which subsequently organize into larger units or microfibrils, eventually resulting in the formation of recognizable cellulose fibers [187], as seen in Figure 10. The crystalline structure is upheld through the cohesive interactions of hydrogen bonds and Van der Waals forces, whereas the amorphous structure is characterized by variabilities in the orientation and arrangement of molecular entities, resulting in twisted or contorted structures.

**Figure 11:**
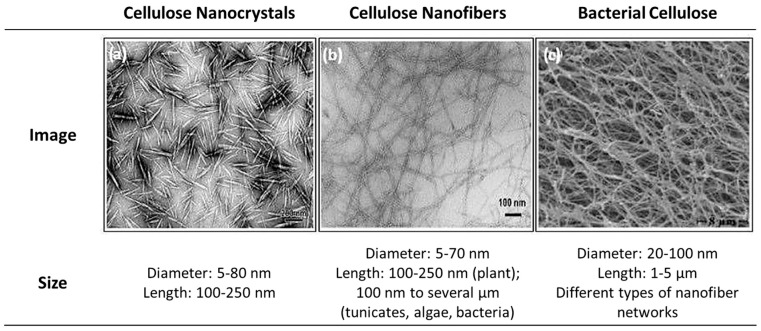
Comparison between the different types of nanocellulose [197].

This polysaccharide has received substantial attention in various forms of cellulose nanomaterials (CN), including cellulose nanocrystals (CNC), cellulose nanofibrils (CNF), and bacterial nanocellulose (BNC) [188], as seen in Figure 10 and Figure 11. Unlike starch nanomaterials, complete standard terms and definitions for CN are proposed in ISO Standard TC 20477 [189]. Applications for CN include reinforcing agents in different nanocomposites; medical applications such as bone tissue engineering [190] or drug delivery [191]; wastewater treatment [192]; and cosmetics [193]. CN obtained from different plants and trees exhibit resemblances between them, although minor differences exist. On the contrary, vast differences in particle shape, size, cross-sectional morphology, and crystal structure can be observed when comparing CN extracted from plants, tunicates, algae, or bacteria against each other [194]. Jonoobi et al. [195] reviewed the different CN preparation methods and reported that even with significant variations in the cellulose content of source materials (20–80%), the resulting purified or bleached cellulose exhibited less diversity in terms of cellulose content, with all measurements exceeding 80%.

##### Cellulose Nanocrystals (CNC)

Cellulose Nanocrystals (CNC), also called nanowhiskers [198], are a versatile class of natural polymer materials with high strength, low density, large specific surface area, high aspect ratio, and hydrophilicity. Moreover, CNC preserve desirable natural cellulose properties like biocompatibility and renewable degradation, with the surface and size effects of nanomaterials [199]. The ISO Standard defines CNC as ‘composed predominantly of cellulose-containing crystalline and paracrystalline regions, with at least one elementary fibril not exhibiting longitudinal splits’, with an ‘aspect ratio (longest to smallest dimension) usually smaller than 50 but usually bigger than 5′ and includes as similarly used terms ‘nanocrystalline cellulose, nanowhiskers cellulose and cellulose crystallites and cellulose microcrystals’ [189].

**Figure 12:**
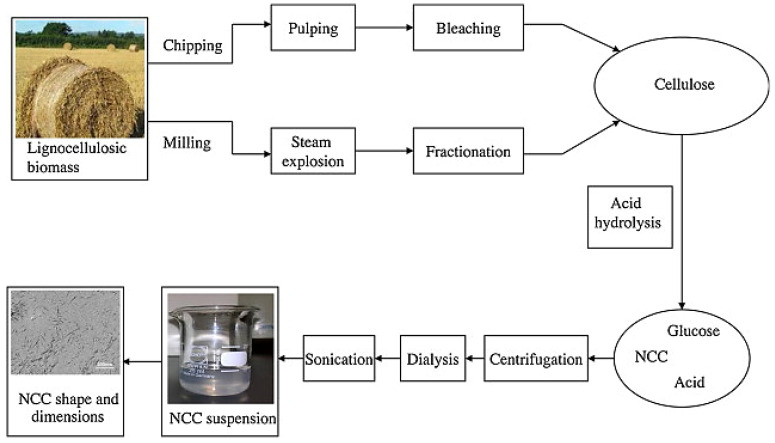
Scheme of main steps for CNC preparation [200].

Similar to starch, cellulose is commonly treated by acid hydrolysis to form CNC, but through a two-step procedure. First, a pre-treatment of the source material is needed to eliminate the hemicellulose, lignin, and other components of the matrix, normally through chemical treatment (pulping) or steam explosion [200]. In the second phase, a hydrolysis reaction is used to destroy the cellulose amorphous fibers and liberate the crystalline region. Usual hydrolysis conditions include a 64–65% (*w*/*w*) sulfuric acid aqueous solution with the ratio of 1:8 to 1:20 (*w*/*v*) of raw material containing cellulose vs. acid at 45 °C, followed by centrifugation washing to a neutral pH, homogenization, and freeze-drying [201], as seen Figure 12. The diversity of dimensions, aspect ratio, morphologies, and degree of crystallinity of CNC are defined by the type of cellulosic source, the conditions during the preparation process, and the experimental methodology employed [187].

The addition of small amounts of wood CNC (1.5–2.5%) to extruded native potato starch formed strong hydrogen-hydroxyl bonds, which improved the mechanical properties while reducing swelling and enzymatic degradation [202]. In contrast, when CNC content exceeded 5 wt%, nanocrystals aggregated, ultimately resulting in a negative impact on physiological conditions [202,203]. The issue of CNC aggregation often arises due to the intermolecular hydrogen bonding interactions that occur between the hydroxyl (OH) groups present on the surface of the nanocrystals [204]. Similarly, beeswax CNC/cornstarch films used to preserve the freshness of fruits displayed oxygen and water barrier capacity and improved tensile strength and modulus [205].

The use of cellulose nanocrystals (CNC) can be impeded by their intrinsic tendency to self-aggregate and hydrophilic behavior. Consequently, several chemical modifications and copolymerization have been documented as strategies to address these deficiencies [206], and to grant additional functions. Some examples include surface modification of CNC through vinyl silane grafting [207]; modified CNC by silane coupling agent and graphene oxide to mechanically reinforce corn starch films [208]; crosslinking CNC by citric acid and amidation of chitosan, which resulted in improved nanocomposites with increased tensile strength, lower water absorption and WVP and good antimicrobial activities against *E. coli* and *S. aureus* [204].

##### Cellulose Nanofibers (CNF)

Cellulose nanofibers (CNF) consist of cellulose chains that are laterally stabilized by strong hydrogen bonds that form between the oxygen and hydroxyl groups of neighboring molecules [195]. The ISO Standard defines CNF as ‘composed of at least one elementary fibril that can contain branches a significant fraction of which are in the nanoscale’, with ‘dimensions of 3–100 nm in cross-section and typically up to 110 µm’, and includes as similarly used terms ‘nanofibrillated cellulose, nanofibrillar cellulose and cellulose nanofibers’ [189]. The diameter of CNF, which can range from approximately 2–20 nm in diameter and extend several microns in length, is primarily influenced by their origin [195]. In contrast to CNC, CNF possess both crystalline and amorphous regions and are generally obtained through a sequence of high-shear mechanical processes of the cellulosic source, mainly high-pressure homogenization, microfluidization, or grinding [156].

Production of CNF using mechanical processes is very energy-intensive; however, pretreatment methods offer an efficient approach to reduce energy input [209,210]. Examples of pretreatment methods include enzymatic treatments [211,212], carboxymethylation [213] and TEMPO oxidation [214]. Pretreatment methods also have a great influence on CNF dimensions; current technology produces CNF with 4–10 nm diameter, while untreated CNF yield coarser fibrils, normally 20–100 nm in diameter, known as cellulose microfibers (CMF) [194]. The downside of these procedures is their relatively high cost and possible unwanted side effects, such as the shortening of CNF length [215]. Moreover, CNF have traditionally been extracted from wood products, but efforts to reduce production costs and increase biomass sustainability have seen several studies detailing the isolation of CNF from alternative sources including agricultural residues and industrial wastes [216,217,218,219].

Nanocomposite films based on potato starch and pineapple leaf CNF (3% *w*/*w*) proved to be UV resistant and have increased water barrier properties [219]. Introducing only 0.4 wt% of henequen CNF in TPS films was enough to increase tensile strength (+80%) and elastic modulus (+170%), and lower WVP (−86%) and oxygen permeability (−94%) [220]. Similarly, extruded starch/wood-CNF composites presented increased mechanical properties and better water sensitivity compared to neat TPS samples, while preserving their translucency [221]. Additionally, all three studies reported that higher CNF concentrations resulted in the aggregation of fibers and lower reinforcement effects.

Chen et al. [222] extracted three kinds of non-wood CNF with the same production method and compared the reinforcement effects on starch-based films. The botanical source had almost no impact on morphology and chemical composition but influenced the size and crystallinity of CNF. The addition of CNF with the highest aspect ratio provided superior mechanical and barrier properties. On a similar note, Zhan et al. [223] compared the effect of adding CNC and CNF to pumpkin starch-based films, revealing CNC provided better reinforcing capacities while CNF showed higher thermal stability. The combination of CNC with clove bud oil (CBO) proved to be an effective additive in starch-based films to preserve red grapes, with CBO further increasing mechanical properties, lowering WVP, and providing antimicrobial properties to the CNC-enhanced films [224].

Biodegradability tests of several nanoparticles in an aqueous environment [225] showed that cellulose and starch NPs exhibited a faster rate of degradation compared to their macroscopic counterparts. Conversely, other nanoparticles with significant and widespread use, such as fullerenes and carbon nanotubes, displayed no detectable biodegradability.

##### Bacterial Cellulose (BC)

In addition to plant cellulose, the production of cellulose can be achieved by means of diverse enzymatic mechanisms, and chemical synthesis from glucose-derived compounds, as well as biosynthesis facilitated by algae, fungi, and bacteria [226]. The cellulose produced by bacterial strains possesses an identical molecular formula and polymeric structure to plant cellulose, with higher levels of crystallinity, and free of lignin, hemicellulose, and pectin [227]. As a result, bacterial cellulose purification and production are easier as there is no need for harsh chemical or high-energy treatments [228]. Moreover, microorganisms can be genetically adjusted to deliver BC with the desired properties and agro-industrial wastes can be utilized as a growth medium to lower production costs [229]. Nevertheless, carbon sources and culture media can have effects on the production and structural properties of bacterial cellulose [230], similar to how the botanical source affects the properties of plant cellulose.

BC is composed of a network of ultrafine nanoscale fibers with extensive polymerization and possesses remarkable physicochemical and mechanical characteristics, comprising high levels of purity, crystallinity, porosity, surface area, translucency, chemical stability, tensile robustness, proton conductivity, and water retention capability [228,231]. Moreover, toxicology studies strongly suggest the use of BC does not pose any adverse side effects when used in human foods [227]. Consequently, BC has been utilized in multiple areas including wound dressing and blood vessel regeneration in the medical field, as biomaterial in electrical instruments, paper restoration, and food ingredients, and as a reinforcement in food packaging [232].

**Figure 13:**
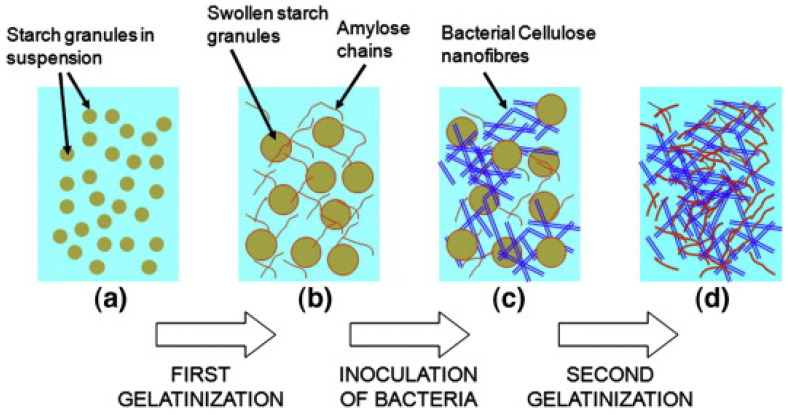
Scheme of the self-assembly BC–starch film: (**a**) Starch granules are in suspension in the culture medium; (**b**) After autoclaving, starch is partially gelatinized, amylose leaches and granules swell; (**c**) BC nanofibrils grow in presence of the partially gelatinized starch; (**d**) After hot pressing, the nanocomposite shows interpenetrating networks of amylose and cellulose. [234].

Melt-mixed corn TPS films containing a 15% bacterial CNC loading prepared by Fabra et al. [233] enhanced oxygen (−95%) and WVP (−46%) barrier properties, preserved transparency, and stiffened the material, with elongation at break quickly decreasing with high loadings. Grande et al. [234] developed bacterial cellulose NF/starch nanocomposites by self-assembly. Unlike previously mentioned methods where cellulose is disintegrated to obtain BCNF, self-assembly starch films capitalize on the inherent ability of certain bacteria to expel cellulose nanofibers and the transport mechanisms of starch gelatinization to form the nanocomposite film, as seen in Figure 13.

#### 3.2.3. Chitin/Chitosan Nanoparticles

Chitin is an abundant biopolymer that originates naturally in exoskeletons of arthropods, cuticles of insects, and in certain fungi and seaweed algae, primarily assuming a supporting function [235]. Chitin nanocrystals can be produced through acid hydrolysis, oxidation (TEMPO-mediated or ammonium persulfate (APS)) and mechanical treatments, while nanofibers can be obtained by a simple grinding process [236].

**Figure 14:**
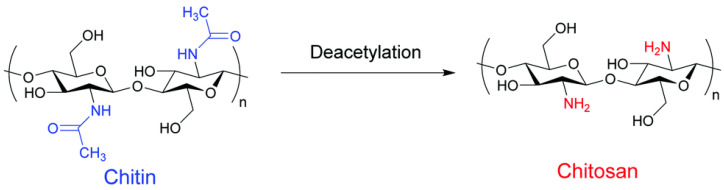
Chemical structures of chitin and chitosan via deacetylation.

Chitosan is a partially or completed deacetylated derivative of chitin, resulting in a linear polysaccharide consisting of (1,4)-linked 2-amino-deoxy-b-D-glucan, as seen in Figure 14. Chitosan offers multiple valuable properties for nanobiocomposite development due to its non-toxic, biodegradable, biofunctional, and biocompatible features, coupled with antimicrobial properties [236,237]. Chitosan nanoparticles can be obtained through various methods including ionic gelation, reverse emulsion, precipitation, and polyelectrolyte complexation [238]. The most promising method for food packaging and pharmaceutical application is ionotropic gelatinization, a physical crosslinking between tripolyphosphate (TPP) and protonated chitosan, that avoids the potential toxicity and undesired effects of chemical crosslink [239,240].

The chemical similarity between the starch matrix and chitin/chitosan NPs creates a strong interfacial interaction, resulting in a reinforcement effect, visible through an increase in tensile strength and decreased elongation at break; an improvement in the intermolecular interaction increasing thermal stability as Tg shifts to higher temperatures; and the creation of a more tortuous path that lowers WVP [241,242]. Additionally, Shapi’i et al. [238] reported that chitosan NP/starch films were more efficient at inhibiting the growth of gram-positive (*B. cereus* and *S. aureus*) bacteria compared to gram-negative (*E. coli* and *S. typhi*) bacteria; a similar conclusion was reached on chitosan NP/tara gum edible films [243].

## 4. Additives

As mentioned before, the properties of materials based on a thermoplastic starch matrix, despite their many benefits, have some well-known drawbacks. Therefore, it is necessary to incorporate additives to improve the material’s intrinsic characteristics or even introduce some new functional properties, such as antimicrobial or antioxidant activity or even some mechanism that can detect the state of degradation of the material [244]. The classification of these additives can be organized into three main groups: plasticizers, essential oils (EOs), and organic molecules.

### 4.1. Plasticizers

A plasticizer is a polymer additive, with a low molecular weight and non-volatile nature, that is added to the polymeric matrix to provide elasticity to the base polymer [245]. Plasticizers increase film flexibility due to their ability to reduce internal hydrogen bonds between polymer chains while increasing molecular space, lowering the glass transition temperature, and increasing the flexibility of the material [246,247]. Moreover, the properties of films can be influenced by the specific plasticizer utilized, as they present varying origins, molecular weights, compositions, and the quantity used, which is normally around 10–40% of dry weight depending on the stiffness of the polymer used [248]. Water is an excellent starch plasticizer but, when removed, films become brittle; therefore, other plasticizers are needed [42]. Common plasticizers in polymeric films include sugars (glucose and sucrose), polyols (glycerol, PEGs, sorbitol, and mannitol), and lipids (phospholipids and fatty acids) [13].

The introduction of plasticizers together with elevated temperatures and shear increases the flexibility of native starches similar to conventional thermoplastic polymers [249]. The most effective plasticizers often resemble the structure of the polymer they plasticize, so the most common plasticizers used in starch-based films are polyols, such as sorbitol and glycerol [247]. During plasticization, polar groups in polyols interact with the hydroxyl groups of starch molecules and reduce internal starch hydrogen bonding. Meanwhile, non-polar groups weaken the van der Waals forces between starch molecules and improve the mobility of starch chains, consequently increasing the tensile properties in films [250]. Consequently, a higher polyol concentration translates into thicker films with lower density, as they increase the disruption and restructuration of the intermolecular polymer chain network creating more free volume [249].

In packaging applications, glycerol is the most utilized plasticizer in starch-based films, due to its stability and compatibility with the hydrophilic bio-polymeric packaging chain [251]. The efficacy of glycerol to generate TPS biodegradable films is largely attributed to its diminutive scale, which grants it enhanced intercalation capabilities between the polymeric chains, and thus confers greater influence over the resulting mechanical properties [252]. Increasing glycerol content has been related to an increase in moisture content, water solubility, water absorption, water vapor permeability, density, elongation at break, lightness, and thickness, while tensile strength and color parameters decreased [253]. As well as a plasticizer for bio-based polymers, this polyol plays a crucial role in another polymer field, particularly as a feedstock to produce synthetic polymers (i.e., polyurethane and polyester) [254]. Interestingly, glycerol is the major by-product of the biodiesel production line, with each cycle of processing yielding around 10% (*w*/*w*) of waste glycerol [254]. Therefore, from a circular economy point of view, consuming glycerol for film production purposes would further promote the sustainability of biodiesel and packaging production.

When comparing glycerol and sorbitol, starch-glycerol films presented lower tensile strength and elastic modulus values and higher elongation compared to starch-sorbitol films, indicating the pronounced plasticizing influence of glycerol [255,256]. Additionally, sorbitol films presented higher thermal stability, as the higher molecular weight of sorbitol decreased the molecular mobility of the polymer chains [256].

Similarly, Maniglia et al. [257] studied the effect of plasticizers on starch-based films from starch obtained through different extraction techniques. Plasticizers with lower molecular weight such as glycerol and urea yielded starch films with higher solubility in water, greater hydrophilicity, higher WVP, and less crystalline structure. On the other hand, plasticizers with a greater number of -OH groups such as sorbitol and glucose were more effective for starches with higher amylose, fiber, and lipid contents and less agglomerated structures.

Ma et al. [250] compared sweet potato starch films with mannitol and sorbitol in different proportions. With the increase in the percentage of sorbitol, more hydrogen bonds formed between the plasticizer and starch molecules, decreasing the tensile strength of starch films. On the other side, higher mannitol content translated to lower freedom of water molecules, limiting the mobility of starch molecules, and thereby resulting in a reduction in water loss and starch regeneration. Finally, the starch film with mannitol:sorbitol (60:40) presented the highest tensile strength, the lowest WVP and was the most effective in delaying retrogradation.

### 4.2. Essential Oils (EOs)

Due to the many characteristics of these additives, essential oils have recently captured the interest of many researchers in the field. In general, essential oils come from a variety of aromatic plant parts, including seeds, bark, flowers, peel, fruit, roots, leaves, and wood. Their biological actions are caused by complex combinations of more than a hundred molecules, such as terpenes and isoprenoids, which are examples of volatile compounds and lipophilic compounds [258]. The EOs are extracted using different techniques such as mechanical extraction, dry distillation, or water hydro distillation that do not include temperature changes [259].

Additionally, EOs have the ability to neutralize a variety of bacteria [260], as well as having antioxidant properties [261]. These oils are particularly interesting additives for their application in thermoplastic starch matrices since they are non-toxic. However, they show some significant drawbacks when used in combination with TPS films. In fact, they tend to decay due to their volatile, unstable composition, limited water solubility, and exposure to light, oxidation, and heat from the outside, among other factors [262]. In order to solve these kinds of issues, encapsulation in polymeric particles [263], liposomes [264] and solid lipid nanoparticles [265] are interesting solutions recently reported in the scientific literature.

**Figure 15:**
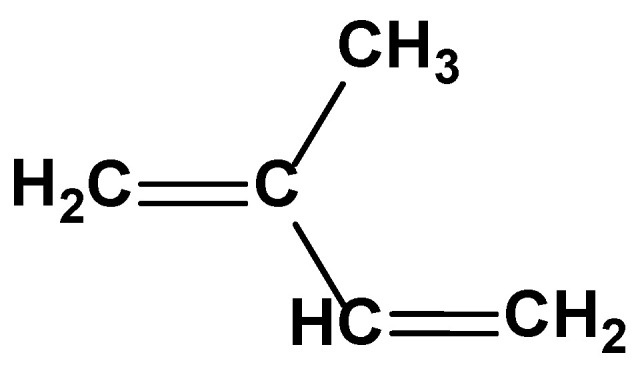
Terpene structure.

- Terpenes: With more than 40,000 distinct chemical structures, terpenes are one of the principal constituents of essential oils and one of the most abundant and diverse categories of plant natural products [266]. There are at least two possible ways to biosynthesize terpenes: from mevalonic acid or from metileritritol phosphate [267]. However, all terpenes are derivates of the isoprene structure (Figure 15).

Hemiterpenes (C5), monoterpenes (C10), sesquiterpenes (C15), diterpenes (C20), sesterpenes (C25), triterpenes (C30), and tetraterpenes/carotenoids (C40) are the different categories of terpenes based on the number of times this structure is repeated [268].

- Polyphenols: These molecules can be divided into different classes depending on their chemical structure. Polyphenols are found in nature as a product of the secondary metabolism of most plants [269]. Two main classes of phenolic acids can be distinguished depending on their structure. Both of them have benzene as a basic bond to a carboxylic group (benzoic acids) or to a propionic group (cinnamic acids), and both structures can be found with different hydroxylation levels [270]. In addition, some examples of molecules inside this group that are not phenolic acids are: coumarins, lignans, chalcones, flavonoids, lignins, and stilbenes [271].

EOs’ constitutions, and therefore their method of action, determine their antibacterial activity [272]. The bacteriostatic or bactericide impact of essential oils is related to their potency [273]. In this regard, the EOs’ efficacy against gram-positive and gram-negative bacteria is very diverse, except for cinnamon, clove, and oregano. The phospholipid bilayer, the membrane proteins, and the degradation of the cell wall by essential oils can enhance the permeability of the cell membrane and cause the loss of cellular components [274].

#### 4.2.1. Rosemary Essential Oil (REO)

Rosemary essential oil (REO) has been widely studied in various matrices, such as PLA. Fiore et al. [275] successfully developed five different biopolymers with the addition of chitosan-based active coatings in which the antioxidant effects of REO delayed the lipid oxidation of raw chicken meat, in comparison to the control sample. In another study [276], active nanocomposite films based on a sodium caseinate matrix were synthesized via the casting method. The films reinforced with ZnO NPs and REO showed better mechanical, physical and barrier properties than the blank films, as well as antimicrobial properties.

Bio-chitosan films were produced via the solvent casting method, using montmorillonite as reinforcement and REO as well as ginger essential oil to add some antimicrobial and antioxidant behavior to the matrix [277]. The measurement of the film properties proved the antioxidant capacity of the essential oils, since the active compounds contained in the chitosan film continued to have the capacity to scavenge the DPPH radical even after casting and migration experiments. Additionally, the films also showed antimicrobial activity against five foodborne pathogens [231].

REO has also been used as an additive in starch-based films. In particular, Restrepo et al. [278] formed edible films using starch as the polymer matrix, enhancing its properties with rosemary and lemongrass essential oils by forming nano-emulsions due to the essential oil’s low water solubility. Edible films were obtained using the solvent casting method, glycerol as a plasticizer (30% *w*/*w* starch) and the EOs’ nanoemulsion concentrations (0.3%, 0.5%, and 1% *w*/*v*). As a result, it was observed that these essential oils caused a plasticizing effect on the matrix that allowed higher transparency and elongation at break.

Moreover, REO has been proven to have high antimicrobial activity against gram-positive bacteria (*S. epidermidis*, *S. aureus*, and *B. subtilis*), gram-negative bacteria (*P. vulgaris*, *P. aeruginosa*, and *E. coli*), and fungi (*C. albicans* and *A. niger*) [279] and to increase antioxidant activity on films coated with REO [275]. The primary components of REO, which constitute 97.41% of the entire oil, are 1,8-Cineole (26.54%), α-Pinene (20.14%), Camphor (12.88%), Camphene (11.38%), and α-Pinene (6.95%), and, according to one hypothesis, the antibacterial properties are a result of their combination [280].

Due to all these antimicrobial characteristics, REO has been one of the most used in starch matrices. In particular, Tafa et al. [281] obtained a nanocomposite of cassava starch, silica nanoparticles, and REO extracted from rosemary leaves, that was prepared to improve the antimicrobial activity, physicochemical properties and water-absorbing and holding properties of starch matrix. 

As mentioned before, REO can act as a plasticizer, allowing for greater transparency and elongation at break, both of which are desired in the food sector and raise water vapor permeation values. However, this impact appears to be offset by the EOs’ hydrophobic properties [278].

#### 4.2.2. Cinnamon Essential Oil (CEO)

Cinnamon essential oil (CEO) is one of the most significant and promising EOs being used in active food packaging due to its antibacterial and antioxidant capabilities. In general, cinnamaldehyde has been primarily involved in CEO’s excellent antibacterial and antioxidant activities. CEO is considered one of the most common EOs in starch matrices for packaging applications because of its great antioxidant properties. In particular, the addition of CEO reduces the moisture content of the films and the water solubility due to its hydrophobic behavior and provides good thermal stability and antioxidant capacity as shown in a recent article where films were obtained via solvent casting, using glycerol as a plasticizer (25% *w*/*w* starch) and varying the bioactive compound [282]. In another study, the addition of CEO in a matrix of starch and sugar palm-based cellulose was studied [283], where an increase in tensile strength and tensile modulus was observed due to the group interactions in the films. Also, the addition of CEO had a plasticization effect on the matrix with a significant increase in the elongation at break and improved ductility of the films [284].

Phenylpropanoid cinnamaldehyde is the primary component of CEO, and its values range from 49.8% to 91% [285,286], followed by Cerpene cinnamyl-acetate [287,288]. Propanol, (+) 3-Carene, Copaene, Eugenol, and α-Pinene are further minor chemicals. Regarding the antimicrobial mechanism, the active ingredients of CEO can go through bacterial membranes and exert their bactericidal effect by acting on a variety of cellular targets. Additionally, they also act on cell physiology causing clumps and self-aggregation on the bacterial membrane [289].

#### 4.2.3. Thyme Essential Oil (TEO)

Thyme essential oil (TEO) is also one of the most promising and widely used essential oils, due to the number of residues that can be used to obtain it and its antibacterial properties against all types of bacteria. TEO has been used in direct contact with foods such as lettuce, meat and milk, showing good results against pathogens of food origin, but less efficacy and less antioxidant activity in food matrices than by itself [290].

Nieto et al. [291], studied the antioxidant capacity of TEO using liposomes as biological membrane model system. To achieve this, after extracting and characterizing the extracted essential oil, they mixed it with a previously prepared liposome solution where peroxidation was induced by adding AAPH in a sodium phosphate buffer (pH 7.4). The reaction was monitored by observing the absorbance at 234 nm, which is the maximum absorption of the conjugated dienes. They obtained promising results on the ability to inhibit the formation of conjugated dienes in a liposome system.

TEO is widely studied as an additive in biodegradable polymers for food packaging, including starch matrices, due to its non-toxic, antimicrobial and antioxidant nature. Cai et al. [292] studied the physicochemical characteristics of a starch film containing TEO microcapsules. Edible TEO films were prepared by coprecipitation technology. In particular, in this process, 30% by weight of glycerol was added as a plasticizer. Samples with different amounts of essential oils, 10%, 20% and 30% by weight of starch and 10% by weight of citric acid, were analyzed. As a result of the study, it was found that the addition of TEO had the ability to inhibit *Botryodiplodia theobromae Pat* and *Colletotrichum gloeosporioides Penz* on the surface of mango fruit. In another study, a starch matrix nanocomposite activated with TEO was observed to improve the shelf life of vegetables, in this case baby spinach leaves. For this, starch/clay nanocomposite films (montmorillonite) and TEO were prepared by the solvent casting method and used as packaging for spinach leaves. As a result, the film could improve the acceptability of leaves by around 8 days by controlling the growth of bacteria such as *E. coli* and *S. Typhimurium* and improving the microbiological quality of baby spinach leaves during refrigerated storage. [293]. However, as the addition of TEO does not affect the mechanical properties, it is usually used with additional reinforcements to enhance mechanical properties [294].

TEO is formed by 52 distinct constituents that have been identified. Moreover, it was discovered that thymol and carvacrol were the 2 most prevalent chemicals along with terpineol, linalool, p-cymene, and other substances [295]. Due to thymol being the primary component of TEO, its antibacterial activity is closely tied to its mode of action. The structural and functional damage to the cell membrane as well as the increase in its permeability are connected to the mechanism of action of thymol. This activity is connected to the loss of inorganic ions, the production of nucleic acids, the decline in membrane potential, protein dysfunction, the collapse of the proton pump, and the depletion of ATP. Before entering the phospholipid bilayer of the cell membrane, it binds to the membrane. Moreover, thymol makes the plasma membrane more permeable to protons and ions, degrades lipid and protein layers, causes the cytoplasm to coagulate and intracellular component leakage, interferes with enzyme systems and cellular metabolism, and finally leads to cell death [296].

**Figure 16:**
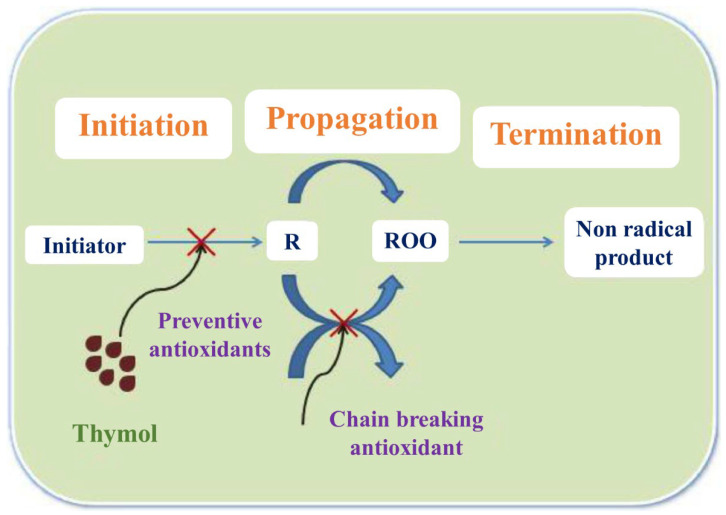
Oxidation mechanism [296].

Aldehydes, ketones, and carboxylic acids are produced at the end of the polymer chain by photooxidation. Antioxidants stop these chain reactions and prevent subsequent oxidation processes by eliminating the free radical intermediates [151]. To perform its antioxidant function, thymol donates electrons to the first hydrogen atom that initiates the oxidation chain [297], as seen summarized in Figure 16.

### 4.3. Organic Molecules

The majority of the components included in this category, such as carvacrol, ferulic acid, and cinnamic acid, can also be found in essential oils, although their usage as single molecules is different [298] or even combined between them [299]. Without the other components that are part of the essential oils, the characteristics of the films that can be obtained directly using the simple molecules can be controlled in a more focused and meaningful way than with the use of the essential oils.

The number of procedures required for the purification of such components is its principal drawback. These molecules are often derived from the essential oils collected from plant resources; as a result, the purifying process entails an increase in overall cost and difficulty.

#### 4.3.1. Carvacrol

**Figure 17:**
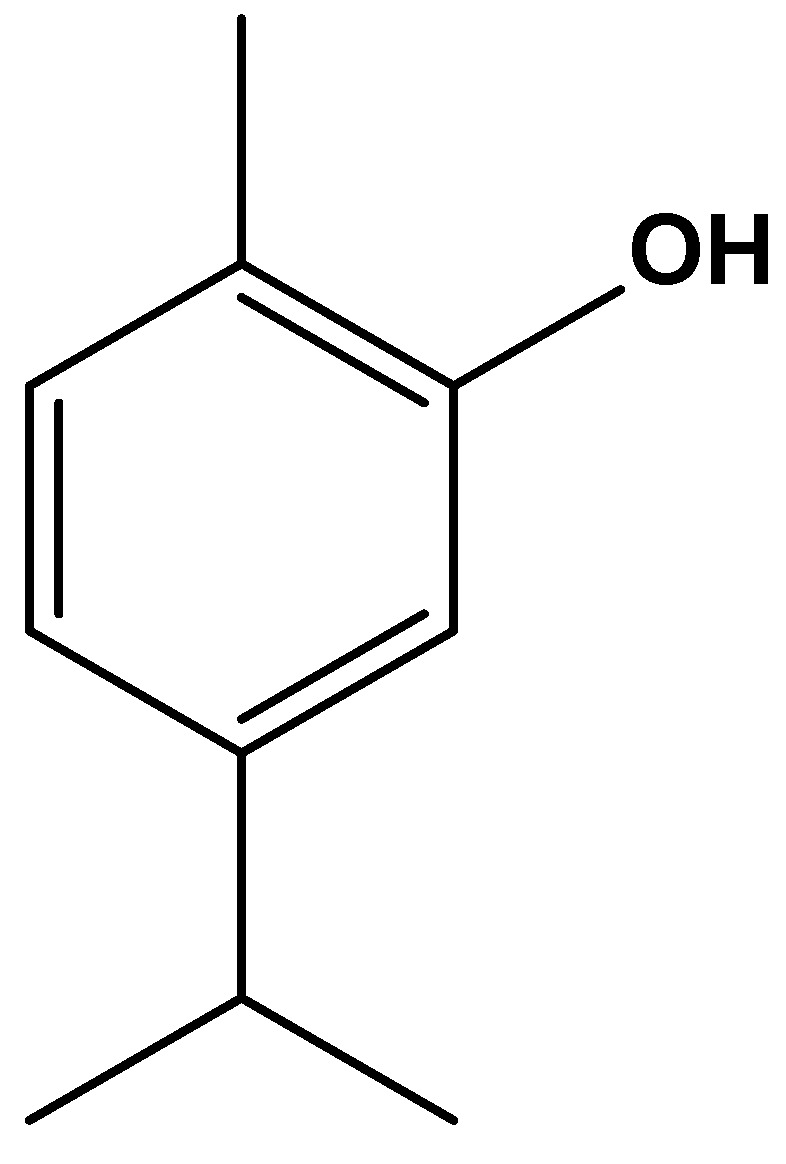
Carvacrol structure.

Carvacrol (Figure 17), a phenol, is the primary component of oregano and thyme essential oils, with a range of 5 to 50% for thyme [295] and between 18–71% in the case of oregano [300,301]. These variances can be due to the different extraction techniques and the parts of the plant that were utilized to extract the molecules.

The antimicrobial activity of carvacrol is well known and studied. Carvacrol has free phenolic hydroxyl groups that can form hydrogen bridges and release protons that can damage the membrane structure and lead to bacterial cell death [302].

Eliuz et al. [303] exposed the carvacrol present in essential oil from *Thymbra spicata* leaves to radiation (at a wavelength of λ > 200 nm), which caused the OH groups to break apart and the H atom released in the orto- or para-position to recombine. However, as different types of cell walls have distinct reactions to UV light, the activity varies between cell walls.

In starch matrices, enhancement of antimicrobial and antioxidant properties via the addition of carvacrol has been widely reported. Edible starch films were obtained using carvacrol and thymol as a method of reducing the incidence of anthracnose symptoms in mango and papaya, enhancing fruit quality as long as possible by an additive effect of both molecules [304]. Mao et al. [305] reported that carvacrol nano-emulsion caused the formation of a porous structure and an increase in the thickness of the film. Moreover, the incorporation of carvacrol decreased the mechanical properties of the nanocomposites, caused by the interactions between carvacrol and the starch matrix via hydrogen bonding.

#### 4.3.2. Eugenol

**Figure 18:**
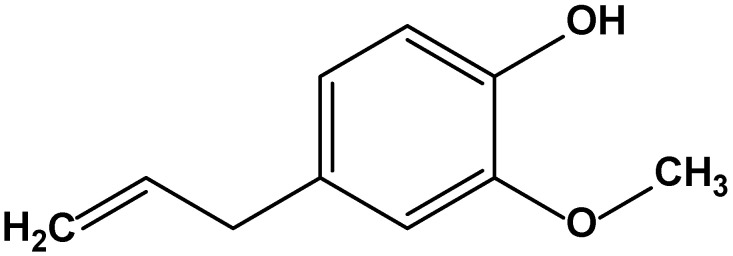
Eugenol structure.

The primary ingredient in some essential oils, such as clove essential oil, which has a phenol content ranging from 76% to 82%, is eugenol (Figure 18) [306,307].

The free hydroxyl groups of this molecule are responsible for most of its antibacterial action, as it can damage cell membranes, increasing nonspecific permeability, which disrupts intercellular ATP and ion transport [308]. Moreover, it has a double bond in the side chain, in the α,β positions, and a methyl group in the position located in the γ position [309]. Due to its hydrophobic properties, it can penetrate the lipopolysaccharide of gram-negative bacteria’s plasma membrane and change the cell structure, which causes intracellular components to infiltrate [310].

The addition of eugenol in starch matrices has been reported in several articles to increase antimicrobial properties. In a study [311], eugenol was encapsulated into 3D-porous starch micro-networks to improve the antimicrobial properties of a corn starch matrix. This system displayed effective antibacterial activities against *E. coli*, *S. aureus* and *B. subtilis* and improved mechanical properties. In a second example [312], eugenol gelatin microspheres showed significantly increased antioxidant capacity and good antibacterial activity.

In relation to the mechanical properties response, these systems show a big dependence on the plasticizer due to the possible hydrogen bond interactions between eugenol and the plasticizer. In particular, Cheng et al. [313] reported that the hydrogen bonds formed between starch and sorbitol showed increased mechanical properties as sorbitol was added into the matrix as well as increased barrier capacity against moisture and oxygen.

#### 4.3.3. Cinnamic Acids and Derivates

**Figure 19:**
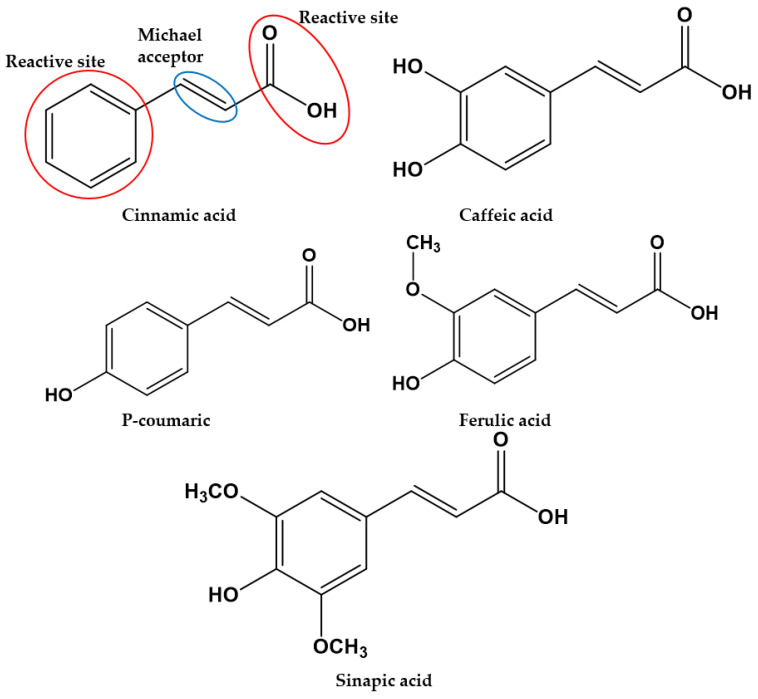
Cinnamic acid and its derivate acids.

Cinnamic acids (CA) and their derivatives (Figure 19) are a wide family of molecules that are usually a minority compound in various types of plants [314,315,316].

The exact molecular mechanisms by which CA acts are unknown. However, two possible mechanisms have been proposed: CA blocks the post-translational modification of proteins that control cell growth, such as some p21ras proteins, thus preventing protein prenylation by blocking the synthesis of residues derived from mevalonate, or CA inhibits the expression of genes that promote cell proliferation [317]. CA exhibits antibacterial efficacy against all gram-negative bacteria, but not against gram-positive bacteria [318].

Moreover, the antimicrobial activity of CA in starch matrices has been reported. In particular, Ordoñez et al. [319] incorporated cinnamic and ferulic acid into starch monolayer films. These films were obtained via the solvent casting method using glycerol as the plasticizer in a ratio of 0.30 g/g starch and 1 and 2% *w*/*w* of ferulic and cinnamic acid, respectively. It was only observed that antimicrobial inhibition in films containing 2% acid was more effective than the ones with just cinnamic acid. There are still no studies that imply any antioxidant effect or modification of mechanical properties by the addition of CA in food packaging applications.

#### 4.3.4. Organic Molecules in Smart Packaging

A new application in food packaging that has been gaining interest from researchers is food safety and quality monitoring, known as smart or real-time packaging. There are three main types of smart packaging, including indicators, data carriers, and sensors. In this regard, food packaging containing an indicator film can detect changes in the quality or freshness of the product, and respond by providing a visual warning to consumers. Briefly summarized, food spoilage can be tracked by alterations in pH as a reaction to the growth and metabolism of microorganisms or the development of organic acids and/or volatile amines (e.g., lactic acid formation in milk or ammonia/amino-sugar formation in meat) [320]. Commonly used synthetic indicators, such as bromothymol blue, methyl red and xylenol dyes, are known to be toxic and cancerogenic to human health [321]. However, natural pH-responsive color-changing indicators offer a simple, non-invasive, non-destructive, inexpensive, and fast-reacting quality-tracking method [321]. Many studies described the preparation of smart packaging films for monitoring food quality by combining biological macromolecules (e.g., starch, cellulose, chitin) with natural colorimetric indicators (chlorophyll, carotenoids, curcumin, betalains, and anthocyanins) [322,323,324].

**Figure 20:**
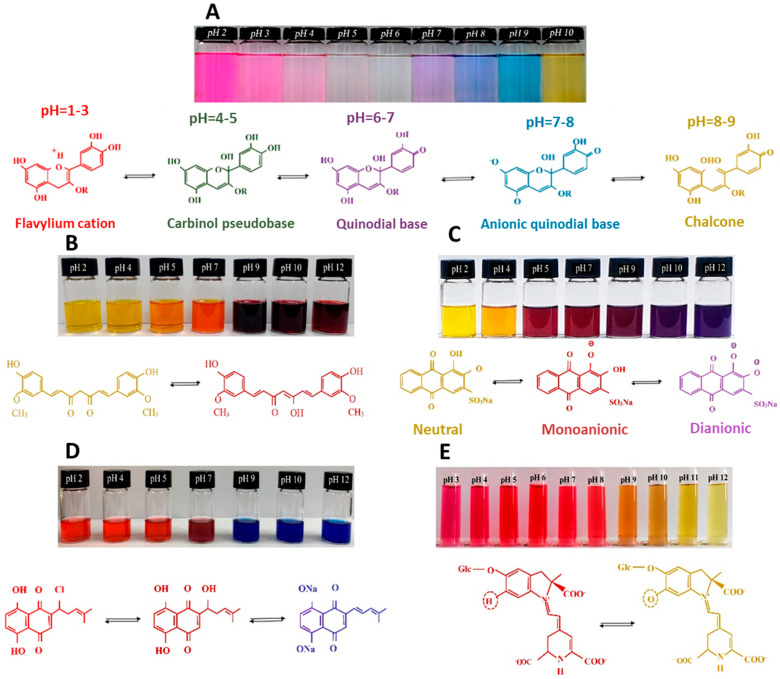
Color changes in response to pH variation of (**A**) anthocyanin, (**B**) curcumin, (**C**) alizarin, (**D**) shikonin, and (**E**) betalains [321].

In terms of natural pH indicators in starch matrices, anthocyanins are by far the most studied, as seen in Table 3. These phenolic compounds can be extracted from a wide variety of fruits and vegetables including red grape, purple corn, black rice, or different kinds of berries [325]. Moreover, anthocyanins are pH-sensitive substances that exhibit a gradient color change for different pH values. Anthocyanins undergo a continuous structural change from flavonoid ions under acidic conditions to quinone structures under alkaline conditions, as seen in Figure 20 [326]. Additionally, anthocyanins also display potent antioxidant activity, which can be useful to prevent color change or lipid oxidation of packaged food products [327].

Fotouhi et al. [328] developed smart starch/gelatin biocomposite films that determined milk freshness and the presence of some preservatives, providing an economical, rapid, and biocompatible diagnostic. The films combined the use of color-changeable films embedded with small anthocyanin, ferric ion, iodide, and diphenyl carbazide analytes with QR codes. Interestingly, Qin et al. [329] studied the effect of encapsulating anthocyanins in starch/PVA blends. When compared to films with unrestricted anthocyanins, the encapsulated films presented denser micro-structures, stronger intermolecular interactions, higher water vapor blocking and mechanical properties, and higher stability when exposed to heat (35 °C) and light radiation.

**Table 3:** Natural pH indicators in starch matrices.

Matrix	Indicator	Botanical Source	Preparation Method	Food Tested	Color Change	Ref.
cassava starch	anthocyanin	grape skin	extrusion	meat	black to purple	[330]
starch + PVA	anthocyanin	roselle calyces	solvent casting	fish	pink to blue to yellow	[331]
agar/potato starch	anthocyanin	purple sweet potato	-	meat	red to green	[332]
starch + gelatin	anthocyanin	purple sweet potato	solvent casting	mushrooms	green to gray to yellow	[333]
native/hydrolyzed cassava starch	anthocyanin	yerba mate	extrusion	-	white to yellow/brown	[334]
sago starch	anthocyanin	red cabbage	solvent casting	-	pink to green to yellow	[320]
starch + gelatin	anthocyanin + [KI/DPC/Fe(III)]	red cabbage	solvent casting	milk	pink to blue to yellow/green	[328]
cassava starch + PVA	encapsulated anthocyanin	black fruit wolfberry	solvent casting	fish	pink to blue to yellow/green	[329]
cassava starch + PVA	betanin	beetroot red	solvent casting	-	red brown to brownish yellow	[335]
potato starch	betacyanin	paperflower	solvent casting	fish	pink to yellow	[336]
corn starch + PVA	curcumin	-	microwave-ultrasound reaction	fish	yellow to red	[337]
TPS + PE	curcumin	-	extrusion	meat	light yellow to light brown	[338]
corn starch	shikonin	zicao roots	solvent casting	shrimps	red to blue	[339]

Besides anthocyanin, curcumin has also been proposed as a pH indicator for smart packaging applications. While curcumin does not possess the wide color range of anthocyanin, it presents a clearer reaction to alkaline environments [337]. Curcumin is a hydrophobic polyphenolic compound, derived from the root of *Curcuma longa,* and is yellow-orange in its natural state [340]. Liu et al. [337] presented starch/PVA based films incorporated with curcumin-loaded Pickering emulsions that presented antioxidant and antimicrobial properties against *S. aureus*, *B. subtilis*, and *E. coli*.

In regards to film elaboration, the use of solvent casting and extrusion techniques has been frequently reported, as seen in Table 3. However, the addition of natural indicators does not solve the mechanical, barrier and processability weaknesses of starch-based films. Consequently, many researchers have often resorted to blends and nanofillers to further enhance film properties for food packaging applications.

## 5. Conclusions and Perspectives

In recent years, biopolymeric materials have been gaining popularity as an alternative to traditional plastic packaging. Moreover, stricter regulations on plastic packaging and the recent interest in the revalorization of agro-alimentary and industrial waste may increase the demand for biopackaging in the near future. Among biopolymers, starch is an abundant, sustainable, and worldwide-available source with interesting properties, that possesses plastic-like behavior in its thermoplastic form. However, the poor mechanical properties and hydrophilic nature make the processed material vulnerable and of poorer quality than its synthetic counterpart.

The integration of nanofillers into starch-based films has been studied and found to enhance several of their physical characteristics, including mechanical properties, thermal stability, moisture resistance, oxygen barrier capabilities, and biodegradation rate. Nevertheless, excessive nanofiller content can lead to negative effects due to the agglomeration and formation of rigid regions within the polymer matrix, and the consequent decline in the interfacial surface area. On the other hand, incorporating additives, plasticizers, essential oils, and organic molecules into the starch films offers interesting functional properties such as antimicrobial, antioxidant or pH-indicative properties. Nevertheless, the addition of nanofillers in nanocomposites is not limited to one element. Multiple research papers have proposed a combination of reinforcement and additives to obtain the benefits of each element while minimizing the downside effects. In summary, from a circular economy point of view, the key point is that thermoplastic starch biocomposites can challenge synthetic packaging when modified with adequate additives and nanofillers.

Therefore, the objective of this review is to discuss starch-based films for food packaging applications. In particular, after analyzing different starch botanical sources and their extraction techniques, the properties of thermoplastic starch are considered, including the main preparation techniques such as solvent casting, extrusion, and film blowing. Then, the most typical organic and inorganic nanofillers used as reinforcements are described, detailing their effects on the mechanical response. Furthermore, additives, plasticizers, essential oils, and functional organic molecules are reported to focus on their antioxidant and/or antimicrobial properties and their application in smart packaging.

## Figures and Tables

**Table 1:** Corn, potato, and rice starch content.

Corn (Maize) Starch		
Content	Amount	Units
Density [26,27,28]	1.356–1.4029	g/cm^3^
Amylose [26,27,28,29]	24.64–29.4	g/100 g
Amylopectin [26,27]	72–75.36	g/100 g
Crude fats [26,27,28]	0.32–7.13	g/100 g
Crude proteins [26,27,28]	7.70–0.38	g/100 g
Moisture contents [26,27,28,29]	10.45–12.7	%
**Potato starch**		
**Content**	**Amount**	**Units**
Amylose [30,31]	17–38.8	g/100 g
Amylopectin [32]	73.42	g/100 g
Crude fats [31]	0.06–0.20	g/100 g
Crude proteins [31]	0.01–0.09	g/100 g
Moisture contents [33]	9–11	%
**Rice starch**		
**Content**	**Amount**	**Units**
Amylose [34,35,36]	4.1–32	g/100 g
Amylopectin [32]	74.56	g/100 g
Crude fats [34]	1.9–28.1	g/100 g
Crude proteins [34]	0–3.0	g/100 g
Moisture contents [34]	13	%

## Data Availability

Not applicable.
